# Fear conditioning is preserved in very preterm-born young adults despite increased anxiety levels

**DOI:** 10.1038/s41598-023-38391-4

**Published:** 2023-07-13

**Authors:** Bilge Albayrak, Lara Jablonski, Ursula Felderhoff-Mueser, Britta M. Huening, Thomas M. Ernst, Dagmar Timmann, Giorgi Batsikadze

**Affiliations:** 1 Department of Pediatrics I and C-TNBS, Pediatric and Developmental Neurology and Center for Translational Neuro- and Behavioral Sciences, University Hospital Essen, University of Duisburg-Essen, Hufelandstrasse 55, 45147 Essen, Germany; 2 Department of Neurology and C-TNBS, Essen University Hospital, University of Duisburg Essen, Hufelandstrasse 55, 45147 Essen, Germany

**Keywords:** Neuroscience, Extinction, Fear conditioning

## Abstract

Very preterm birth is associated with an increased risk for anxiety disorders. Abnormal brain development may result in disordered fear learning processes, which may be exacerbated by environmental risk factors and persist in adulthood. We tested the hypotheses that very preterm-born young adults displayed higher levels of fear conditioning, less differentiation between threat (CS+) and safety (CS−) signals, and stronger resistance to extinction relative to term-born controls. A group of 37 very preterm-born young adults and 31 age- and sex-matched term-born controls performed a differential fear conditioning paradigm on two consecutive days. Acquisition and extinction training were performed on day 1. Recall and reinstatement were tested on day 2. Preterm-born participants showed significantly higher levels of anxiety in the Depression-Anxiety-Stress-Scale-21 questionnaire. The fear conditioning outcome measures, skin conductance response amplitudes and anxiety ratings, were overall higher in the preterm-born group compared to controls. Awareness of CS-US contingencies was mildly reduced in preterms. Acquisition, extinction, recall and reinstatement of differential conditioned fear responses (CS+  > CS−), however, were not significantly different between the groups. There were no significant group by stimulus type interactions. The finding of largely preserved associative fear learning in very preterm-born young adults was unexpected and needs to be confirmed in future studies.

## Introduction

Substantial progress has been made in obstetrical and neonatal care of very preterm-born infants over the last decades^1^. As a consequence, the rate of mortality and and severe morbidity leading to cerebral palsy has been significantly lowered^2,3^. However, cognitive impairments as well as emotional and behavioral problems have become more apparent, and are reported in up to 50% of very preterm-born children and adolescents^4^. Following the vulnerability hypothesis, mental disorders with early manifestation in childhood and adolescence are a frequent progenitor of mental disorders in adulthood^5^. Prematurity is a risk factor for emotional and behavioral disorders throughout life, and the relative frequency seems to be increasing^4^**.**

More specifically, very preterm-born children and adolescents have a significant higher risk for attention-deficit disorder with and without hyperactivity (ADD/ADHD) and autism spectrum disorders (ASD) compared to their term-born peers^6–9^. Furthermore, very preterm birth (≤ 32 weeks of gestation) is associated with a 2–4 times increased risk for emotional problems such as anxiety disorders, depression, and obsessive–compulsive disorders^9–14^. The co-occurrence of symptoms associated with ADD/ADHD, ASD and anxiety disorders has been denoted as “preterm behavioral phenotype”, and occurs mainly in children with very low birth weight^15^.

Aberrant brain development likely plays a role associated with diffuse white matter disease, a thin corpus callosum, enlarged lateral ventricles and a reduction of cerebellar volume^16^. This aberrant brain development has been summarized under the term “encephalopathy of prematurity” by Volpe^16^. Environmental factors may also contribute to the development of emotional and behavioral problems. Very preterm-born infants experience a stressful peri- and postnatal period with repeated painful procedures, sleep interruptions, an artificial environment and a long-lasting separation from their parents^17–19^.

Abnormal learning processes are thought to play an important role in the development of mental disease^20^. For example, anxiety disorders are frequently related to disordered acquisition and extinction of conditioned fear responses^21^. It is conceivable that the abnormal brain development results in disordered emotional learning processes, which are exacerbated by environmental risk factors and persist into aduldhood. Initial studies in rodent models of prematurity have reported altered fear conditioning^22–24^. Freezing responses have been found to be increased to the context in a fear conditioning task in a rat model of preterm neonatal anoxia^23^, whereas Pierre et al.^24^ reported reduced freezing responses to the auditory cue in acquisition training and retrieval in a rat model of inflammation induced neonatal white matter injury. Repetitive pain application in neonatal rats resulted in reduced freezing responses in a trace and a contextual fear conditioning task^22,24^.

Furthemore, a reduced volume of the amygdala and hippocampus have been shown in very preterm-born children and young adults, important hubs of the fear conditioning circuitry^25,26^. As yet, it is unknown whether young adults born preterm have deficits in the acquisition and extinction of learned fear responses. Based on previous findings in patients with anxiety disorders, we tested the hypotheses that very preterm-born young adults display higher levels of fear conditioning^27^, less differentiation between the CS+ (that is, threat) and the CS− (that is, safety) signals^28^, and stronger resistance to extinction relative to healthy controls^29^.

## Results

### Depression-Anxiety-Stress-Scale-21 (DASS-21) questionnaire

**Figure 1:**
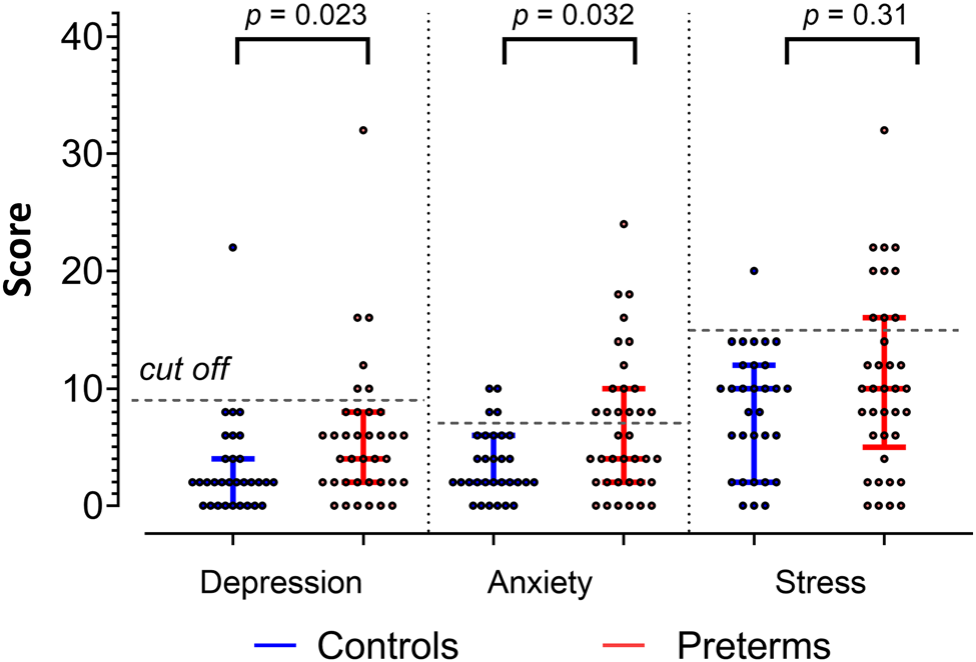
Depression-Anxiety-Stress-Scale-21 (DASS-21) questionnaire. Median scores and interquartile range (IQR) in the preterm and control groups. Dots represent individual scores. Normal range: depression score: 0–9, anxiety score: 0–7, stress score: 0–14; maximum possible score: 42^30^.

Scores differed between preterm and control groups especially in the anxiety scale (Fig. 1). Sixteen (43.2%) preterm participants showed mildly to moderately elevated scores of anxiety, but only four (12.9%) control participants. Nine of the twelve preterm participants, who had a history of emotional disorders, were part of the subgroup with elevated anxiety scores. Six (16.2%) preterm participants and one (3.2%) control presented with mildly elevated depression scores. Ten (27%) preterms and one (3.2%) control showed mildly elevated stress scores. Anxiety (U = 399.5, *p* = 0.032, Mann–Whitney-U test) and depression scores (U = 388.5, *p* = 0.023) were significantly higher in the preterm group compared to controls. Stress scores were not significantly different between groups (U = 490, *p* = 0.31).

### Skin conductance responses

**Figure 2:**
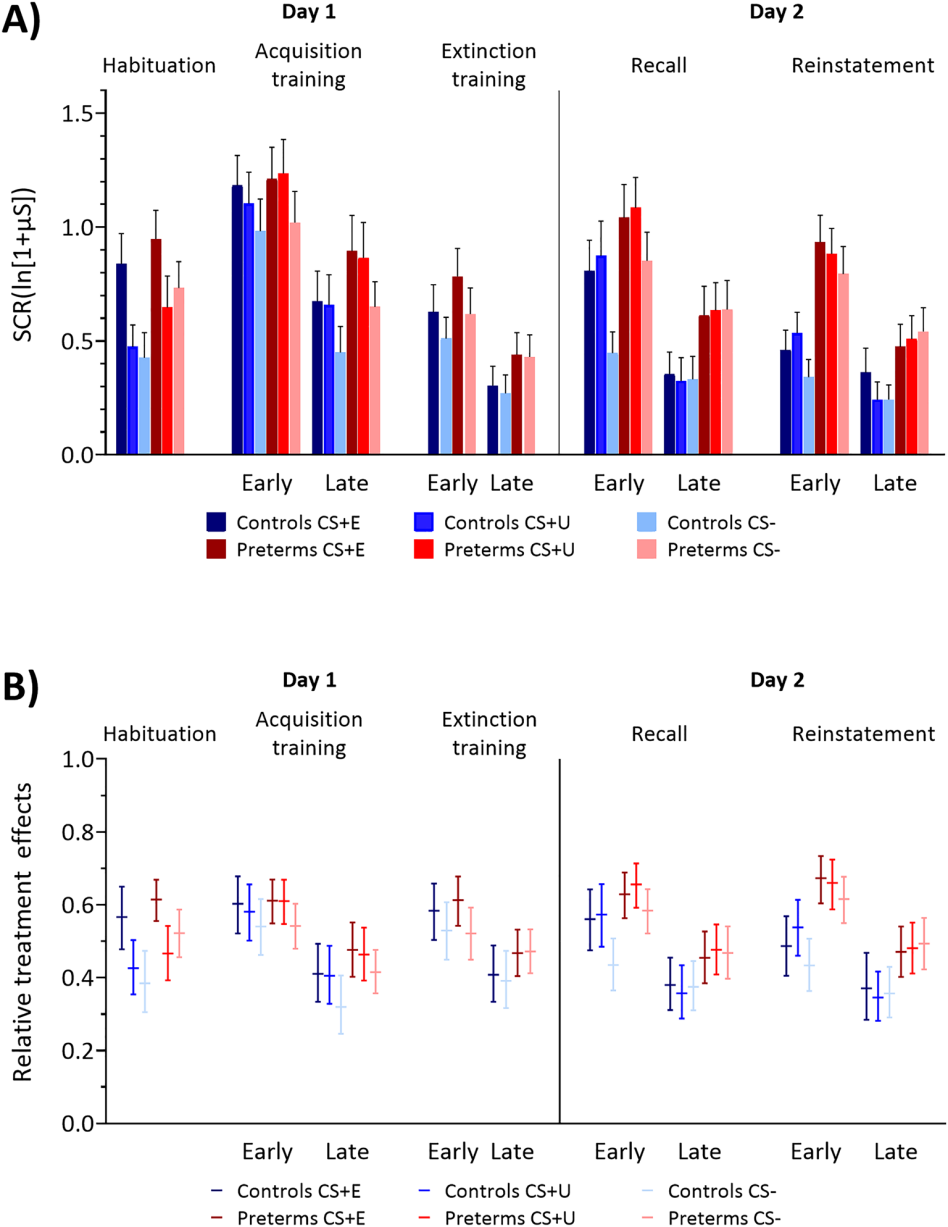
(**A**) Skin conductance response amplitudes and (**B**) respective relative treatment effect (RTE) estimates. (**A**) Colored bars represent group mean (log-transformed) values for habituation phase and early and late blocks of acquisition training, extinction training, recall and reinstatement phases. Error bars indicate S.E.M. (**B**) Horizontal lines denote median RTEs and whiskers denote 95% confidence intervals. Blue colors = controls, red colors = preterms. Dark colors: CS + E and CS + U, light colors: CS-.

Group mean SCR amplitudes in the habituation phase, early and late acquisition and extinction training on day 1, and early and late recall and reinstatement phases on day 2 are shown in Fig. 2. Statistical findings are summarized in Table S1 in Supplementary data. Throughout the experiment, mean SCR amplitudes were numerically higher in the preterm group (red columns) compared to the control group (blue columns). The group difference was significant in reinstatement phase. No other significant difference between the preterm and control groups was observed.

Exploratory analysis of non-zero SCRs indicated no statistically significant differences between non-zero SCRs in the preterm group compared to the control group (Table S2 in Supplementary data) with the exception of the reinstatement phase (*F*_(1,64.3)_ = 5.17, *p* = 0.026, one-way non-parametric ANOVA-type statistic).

#### Habituation phase (day 1)

Mean SCR amplitude was higher towards the CS + E compared to the CS + U and CS− in both groups (Fig. 2A; Table S1). The reason for this difference was that the CS + E was always shown first, and both preterms and controls showed a significantly higher SCR amplitude to this very first trial compared to all other habituation trials. Importantly, at the end of the habituation phase there was no significant difference in SCR amplitude between the three stimuli (CS + E, CS + U and CS−) (see Fig. S1, Table S3 in Supplementary data). SCRs in the first CS + E trial were significantly higher than CS + U and CS− (all *p* values < 0.001, least squares means test) in both groups, but not in the second trial (all *p* values > 0.999, least squares means test).

#### Acquisition training (day 1)

Both groups learned to differentiate the CS + U and the CS + E from the CS− in the acquisition training with significantly higher SCR amplitudes towards the two CS + s compared to the CS− (Fig. 2A, Table S1 in Supplementary data; see also Fig. S1 and Table S3 for individual trial data). This difference was more prominent during late acquisition in both groups. As expected, SCR amplitudes declined in late compared to early acquisition, with SCR habituation being a common finding in fear conditioning studies^31–33^. Non-parametric ANOVA-type statistics revealed significant main effects of stimulus (CS + E vs CS + U vs CS−: *F*_(1.77,108)_ = 16.66, *p* < 0.001) and block (early vs late phase; *F*_(1,58.1)_ = 90.37, *p* < 0.001), but not of group (*p* = 0.482). None of the interactions were significant: block × stimulus (*p* = 0.886), group × stimulus (*p* = 0.886), group × block (*p* = 0.090), group × stimulus × block (*p* = 0.504).

#### Extinction training (day 1)

At the beginning of the extinction training, SCR amplitudes were higher towards the CS + E compared to the CS− in both groups, confirming that participants had learned to differentiate between the CS + E and CS− in the acquisition training. Both groups learned to extinguish the conditioned responses, with no significant difference between the CS + E and CS− in late extinction. Non-parametric ANOVA-type statistics revealed significant main effects of block (early vs late phase; *F*_(1,62.6)_ = 29.79, *p* < 0.001), stimulus type (CS + E vs CS−; *F*_(1,61.5)_ = 12.35, *p* = 0.001) and a significant block × stimulus (*F*_(1,55.7)_ = 7.59, *p* = 0.008) interaction. The group main effect (*p* = 0.513) and all other interactions were not significant: group × stimulus (*p* = 0.730), group × block (*p* = 0.210) or group × stimulus × block (*p* = 0.236). In early extinction, SCRs in CS + E were significantly different from CS− (*p* = 0.001, least squares means test) and SCRs to both CSs were significantly higher in early than in late block in both groups (all *p* values < 0.005, least squares means test).

#### Recall phase (day 2)

In early recall, SCR amplitudes were higher in CS + E and CS + U blocks compared with CS− blocks in both groups. In late recall, SCR amplitudes did not differ between CS + s and CS− in both groups. Non-parametric ANOVA-type statistics revealed significant main effects of block (early vs late phase; *F*_(1,64.6)_ = 56.42, *p* < 0.001) and stimulus type (CS + E vs CS + U vs CS− ; *F*_(1.87,116)_ = 6.93, *p* = 0.002) and a significant block × stimulus interaction (*F*_(1.81,106)_ = 8.82, *p* < 0.001). The group main effect (*p* = 0.098) and all other interaction effects were not significant [group × stimulus (*p* = 0.223), group × block (*p* = 0.922) or group × stimulus × block (*p* = 0.235)]. In early recall, SCRs in CS + E and CS + U blocks were significantly different from CS− block in both groups (all *p* values < 0.001, least squares means test). No difference between CS + E and CS + U were revealed (*p* values = 1.0, least squares means test). In early recall, SCRs were significantly higher compared to the late block (all *p* values < 0.002, least squares means test).

#### Reinstatement phase (day 2)

SCR values were higher in the preterm group compared with controls towards all CSs. In early reinstatement, SCR amplitudes were numerically higher towards the CS + s compared to the CS− in both groups. These differences did not survive Bonferroni correction for multiple comparisons (all *p* values > 0.062, least squares means test). Non-parametric ANOVA-type statistics revealed a significant main effect of group (preterm vs control; *F*_(1,65.9)_ = 6.96, *p* = 0.01). Main effects of block (early vs late phase; *F*_(1,63)_ = 38.28, *p* < 0.001) and block × stimulus interaction (*F*_(1.88,104)_ = 3.89, *p* = 0.026) were significant. No significant main effect of stimulus (*p* = 0.148), and no significant interaction effects [group × stimulus (*p* = 0.660), group × block (*p* = 0.414), group × stimulus × block (*p* = 0.287)] were revealed. SCRs in preterm group were significantly higher compared to SCRs in the control group throughout the whole reinstatement phase (*p* = 0.01, least squares means test). In early reinstatement, SCRs to all CSs were significantly higher compared to the late block (all *p* values < 0.004, least squares means test).

### Questionnaires

#### Valence, arousal, fear

**Figure 3:**
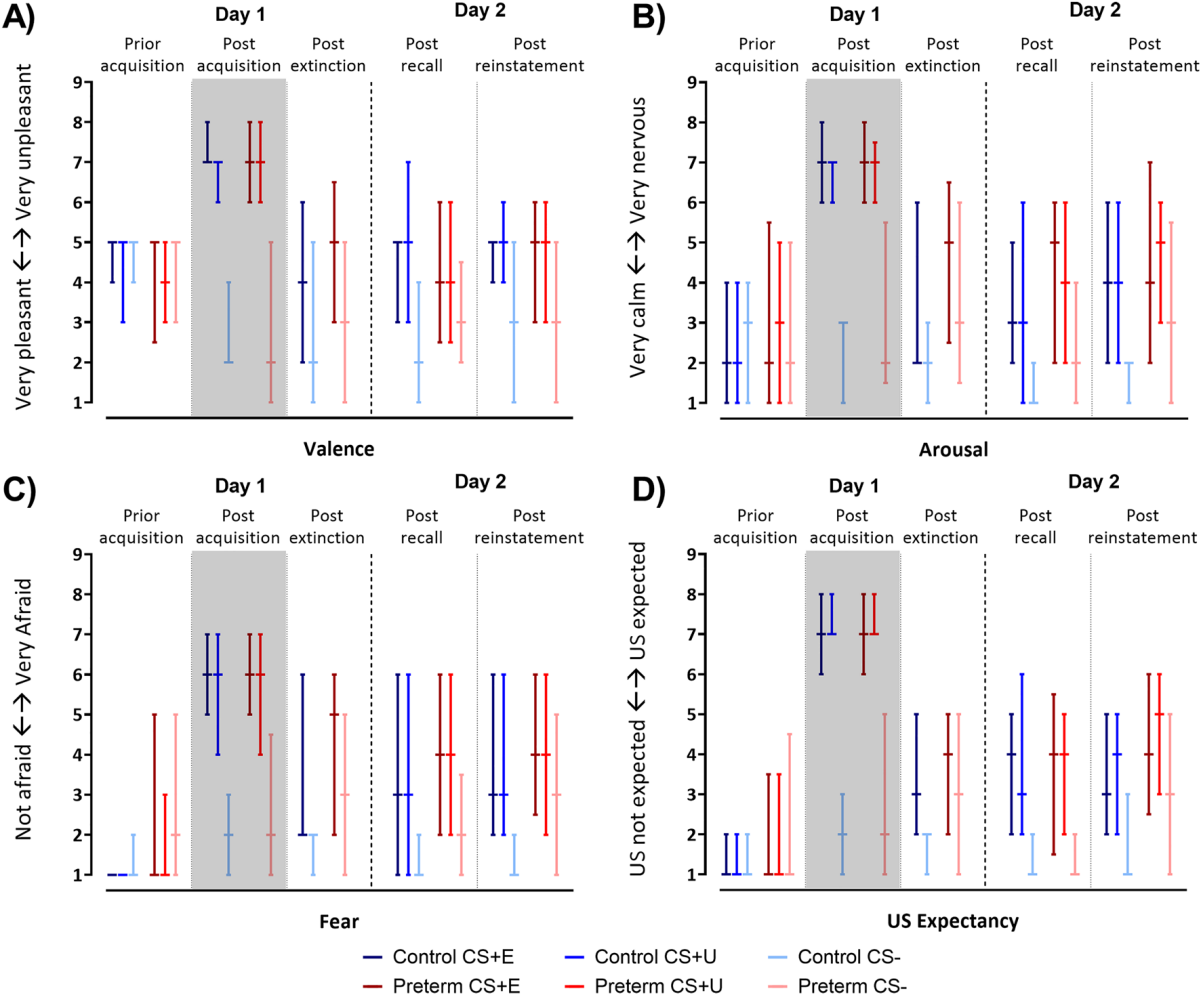
Median ratings regarding (**A**) valence, (**B**) arousal, (**C**) fear and (**D**) US expectancy on a Likert-scale of 1 (*“very pleasant”/“very calm”/“not afraid”, “US not expected”*, respectively) to 9 (*“very unpleasant”/“very nervious”/“very afraid”, “US expected”*, respectively). Horizontal lines denote median values. Whiskers range from the first to the third quartile. Blue colors = controls, red colors = preterms. Dark colors: CS + E and CS + U, light colors: CS−. Gray background = fear acquisition training. For respective relative treatment effects see Fig. S2 in Supplementary data.

Prior acquisition training there was no significant difference in ratings of the CS + s and the CS- in both groups. Post acquisition training valence of the CS + s was rated significantly less pleasant compared to the CS−, arousal and fear were rated significantly higher (Fig. 3A–C, all *p* values > 0.001, see Table S1 for summary of non-parametric ANOVA-type statistical analysis). Differences between CS + s and CS− ratings remained post extinction, post recall and post reinstatement.

Post hoc tests showed significantly less pleasant valence rating, significantly higher arousal and fear ratings towards CS + E and CS + U compared to CS− post acquisition, post extinction training, post recall and post reinstatement (least squares means tests, all *p* values < 0.001), but not prior acquisition training (least squares means test, all *p* values > 0.074). Main effects of time (prior acquisition vs post acquisition vs post extinction vs post recall vs post reinstatement) were significant (all *p* values < 0.001). Regarding fear ratings the main effect of group was significant (controls vs preterms: *F*_(1,65.8)_ = 3.92, *p* = 0.048). Main effect of group for fear rating indicates overall higher levels of fear that preterm participants.

#### US unpleasantness, US expectancy, CS-US contingency

Preterm and control participants rated the US as unpleasant and unpleasantness was not different between groups [post acquisition training: preterms: median 7 (IQR 7–8); controls: median 8 (IQR 7–8); on a Likert scale from 1 (not unpleasant) to 9 (very unpleasant); Mann–Whitney U test: U = 482.5, *p* = 0.347].

US expectancy, as an index of conscious CS-US contingency awareness^34^, was not different between groups. Both groups learned the CS-US contingencies.

Prior acquisition training, US expectancy ratings after CS + E, CS + U and CS− presentations were not different. Post acquisition US expectancy after the CS + E and CS + U were rated significantly higher compared to the CS− (all *p* values < 0.001). No significant differences between groups were revealed post acquisition, extinction, and recall (all *p* values > 0.221; non-parametric ANOVA on individual time points). Post-reinstatement US expectancy ratings were significantly higher in preterms than in controls (*p* = 0.03; Fig. 3D, Table S1).

**Figure 4:**
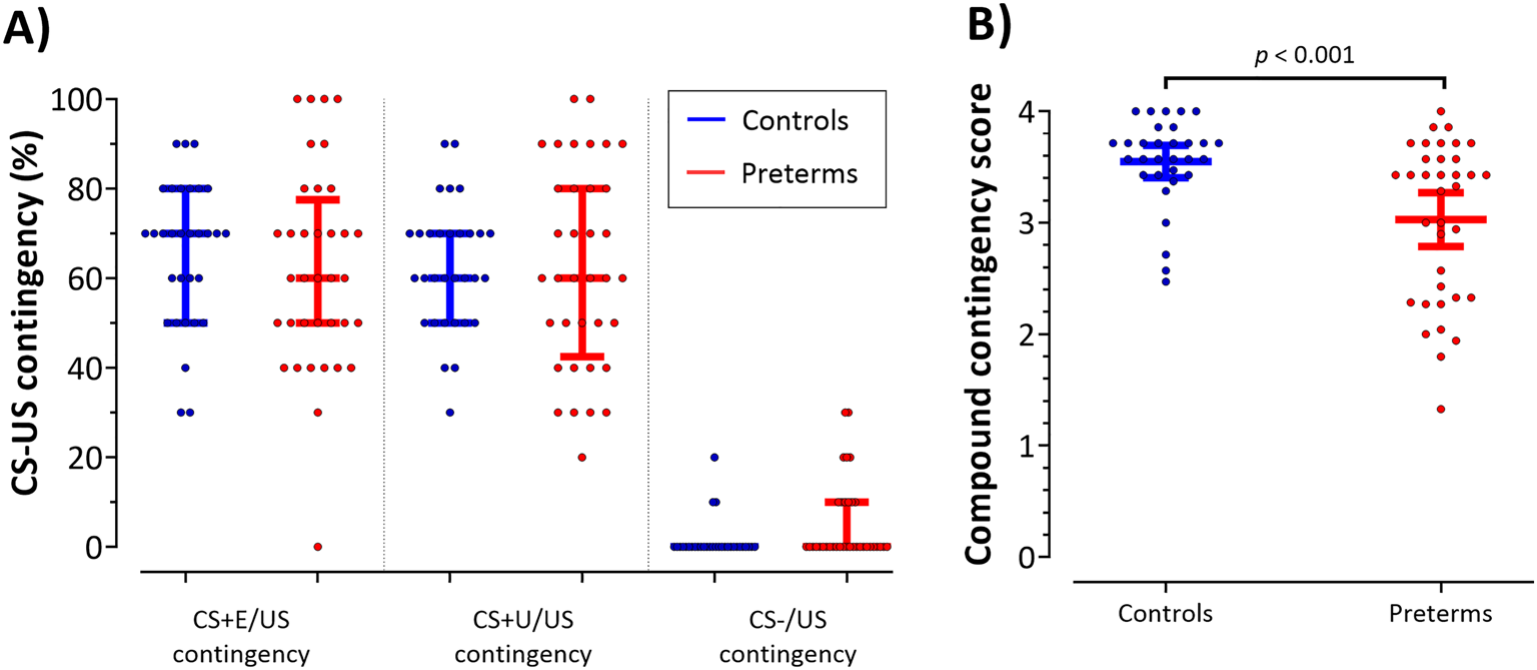
CS-US contingency assessed after the acquisition training. (**A**) Group median reported CS-US contingency and individual data. Horizontal line represents median value, whiskers range from the first to the third quartile. (**B**) Group mean compound contingency scores and individual data. Horizontal line represents mean value, error bars indicate 95% confidence intervals. Dots represent individual data. Blue color = controls, red color = preterms.

Post acquisition training, one control (3.2%) and nine preterms (24.3%) reported that they did not recognize a pattern between CS+ and US presentations. The rest of participants reported that they recognized a pattern between CS+ and US contingency after 3.76 ± 2.36 min. There were no significant differences between groups (Mann–Whitney U test; U = 327, *p* = 0.150). To analyze in more detail whether learning of the CS-US contingencies differed between control and preterms, who recognized a pattern, a compound contingency score was calculated considering the following four questions, which were asked at the end of the acquisition training: (1) estimate the percentage of US after CS + E; (2) estimate the percentage of US after CS + U; (3) estimate the percentage of US after CS; (4) which color (CS) was never followed by an US. The subscores for the first three questions were calculated by how far they were from the correct answer in 10% steps and were rated between 1 (most accurate) and 0 (most inaccurate). The question 4 had a binary 0 or 1 score, as there were only correct and incorrect answers. The overall compound score is the sum of the above subscores and ranged from 0 (wrong answer to all questions) to 4 (correct answer to all questions). Preterm born participants scored significantly below controls concerning the calculated compound contingency score (Fig. 4; non-parametric one-way ANOVA, *F*_(1,65)_ = 13.54, *p* < 0.001).

The effects of IQ on questionnaire data were analyzed with IQ as a covariate. Results remained unchanged (see Table S4 in Supplementary data).

### Comparing anxious and non-anxious preterm participants

In addition, we tested whether preterm participants who scored in the DASS-21 anxiety subscore in the abnormal range performed differently from preterms who scored within the normal range (16 anxious and 21 non-anxious preterm participants based on a cut off > 7; see Fig. 1). The control group consisted of the 27 participants who scored within normal range (non-anxious control group), with four controls performing above. Data is presented in Supplementary data. Subgroup analyses did not reveal any effects of anxiety levels in preterm participants on any measure (Figs. S3–S7; Tables S2–S6).

### Comparing males and females

Finally, we tested whether sex had an effect on the results. The analyses were redone considering sex as a covariate. For none of the comparisons, sex as a covariate became significant (Table S7 in Supplementary data). Furthermore, statistical findings with and without sex as a covariate remained largely the same, indicating that sex did not significantly influence the observed group differences (compare with Table S1 in Supplementary data).

## Discussion

The aim of the present study was to evaluate fear conditioning in very preterm-born young adults, a patient group at risk for the development of anxiety disorders. Acquisition and extinction learning was largely preserved, except for slightly limited learning of the CS-US contingencies. Recall and reinstatement of learned fear responses after successful extinction training were not significantly different from term-born young adults. Anxiety ratings and skin conductance amplitudes were generally higher in very preterm-born young adults, but these findings were unspecific and not linked to aberrant fear-related associations.

### Preterms show high levels of anxiety

A higher percentage of the very preterm-born young adults than controls showed elevated depression, anxiety and stress scores in the Depression-Anxiety-Stress-Scale-21 (DASS-21). Our observations that very preterm-born young adults are more anxious match well with the literature as outlined in the introduction^9,35–37^. Increased anxiety levels in the DASS-21 make it less likely that the largely negative findings of the present study are explained by a lack of anxious phenotype in our study population. Furthermore, results did not change when only the very preterm-born subgroup who rated within the abnormal range in the anxiety subscore of the DASS-21 was investigated.

### Acquisition and extinction of conditioned fear responses is preserved in preterms.

We tested the hypotheses that very preterm-born young adults display higher levels of learned fear responses to threat cues, and less differentiation between the threat and safety cues. In both cases, a significant interaction between conditioned stimulus type (CS+, CS−) and group is to be expected. This, however, was not the case. Both the very preterm-born and the term-born groups showed significantly higher fear responses towards both CS + s as compared to the CS− in the early and late acquisition training. This difference remained in early extinction training and vanished during late extinction. However, there were no significant stimulus type by group, or stimulus type by group by block interaction effects, neither considering SCR amplitude nor ratings of fear, arousal, and valence. SCR amplitudes appeared generally increased in preterms including the initial habituation phase, but the group effects did not become significant. Likewise, fear ratings were generally higher in preterms compared to controls.

The lack of significant abnormalities in the acquisition and extinction of differential conditioned fear responses in very preterm-born young adults was unexpected. Negative findings, however, are in accordance with metanalyses of fear conditioning studies in patients with different anxiety disorders showing that initial findings are frequently difficult to replicate and abnormalities are frequently weak^21,38^.

Findings of preserved acquisition of conditioned fear responses are also at variance with a previous study of our group showing a significantly reduced ability to acquire conditioned eyeblink responses in very preterm-born children and young adults^39^, another form of aversive associative learning. Delay eyeblink conditioning is strongly cerebellar dependent and prematurity has a significant detrimental effect on the cerebellum^40–42^. Important developmental processes of the cerebellum are interrupted by prematurity leading to cerebellar growth failure^17–19^. The cerebellum, on the other hand, is not only involved in eyeblink conditioning, but also in fear conditioning^43,44^. However, fear conditioning relies on a more widespread fear learning circuitry including the amygdala^45–47^. Amygdala and related fear circuitry are likely to be also affected by prematurity^18,25,26^, but possibly to a lesser extent as the cerebellum^16,19,48^. Likewise, fear conditioning is only mildly affected in cerebellar disease, and significantly less compared to the detrimental effects on eyeblink conditioning in humans^49^.

### Awareness of CS-US contingencies is reduced in preterms.

Mean US expectancy ratings towards the CS + s und CS− were not significantly different comparing preterms and control participants. The percentage of preterms, however, was less who reported that they recognized a pattern between the CS + and US presentation after the acquisition training. Furthermore, in preterms who recognized a pattern, contingency ratings were significantly less accurate compared to controls. Thus, knowledge of the CS-US contingencies was less in very preterm-born young adults at the end of acquisition training. Contingency awareness is likely linked to attention and working memory processes^50^. Very preterm-born participants have a high risk of attention and learning problems^7,9^ which has been associated with cerebellar growth failure^51,52^. Awareness of the CS-US contingencies is considered a prerequisite for conditioned skin conductance responses to occur^34^. Interestingly, Baas et al.^53^ showed that failure to become aware of the CS-US association resulted in higher contextual fear, and that those participants who remained unaware tended to exhibit higher trait anxiety. This may at least partially explain the higher anxiety scores and increased level of SCRs throughout the experiment in the preterm-born group compared to controls^53^.

The observed deficits in awareness, however, were small. The known variability of SCRs may have prevented the detection of mild fear conditioning deficits in preterms. The observation of reduced CS-US contingency awareness warrants future studies in larger preterm populations.

### Unaltered recall and reinstatement of learned fear following extinction in preterms.

Extinction learning does involve more than erasure. There is new inhibitory learning going on, that is participants learn that the CS+ is no more followed by an aversive stimulus^54^. A stronger resistance to extinction relative to healthy controls is thought to contribute to the development of anxiety disorders^29^. Extinction learning, however, was not significantly different between preterms and controls.

Phenomena like spontaneous recovery, renewal and reinstatement provide evidence that the initial fear memory is not erased during extinction learning, and are possible reasons why exposure therapy shows frequently return of fear^55,56^. During tests of recall and reinstatement, there is competition between the learned extinction memory and the learned fear memory^57,58^. Return of learned fear following extinction learning has been shown to be stronger in patients with anxiety disorders compared to controls^58–60^.

In early recall, both preterms and controls showed spontaneous recovery, that is, a return of differential fear responses, with higher fear responses to the CS+ compared to the CS−, which vanished in late recall. Neither controls nor preterms showed a significant difference between the CS+, which had been extinguished and the CS+ which had not been extinguished. Thus, both groups showed high levels of spontaneous recovery of learned fear towards the extinguished CS+. Participants were instructed on day 1 that should they perceive a pattern between stimuli, the experimenter would not change that pattern during the experiment. The instruction was chosen to allow for robust acquisition learning which is a prerequisite to access extinction learning^34^. It may explain why recall of the initial fear association was stronger than recall of the extinguished responses towards the CS + E. Similar to acquisition and extinction, SCR amplitudes were numerically higher in preterms. There were, however, no significant stimulus type (CS + E vs CS + U vs CS−) by group interactions. Comparable results were obtained in testing of reinstatement effects, that is reinstatement of fear responses after (unpaired) US presentation following the renewed extinction of fear responses. There were no significant differences in SCR responses towards the CS + s and the CS−, that is, we saw generalized reinstatement effects (in both groups) which is frequently observed in humans^61^. Furthermore, the general increase of SCR responses in preterms compared to controls observed throughout the experiment became significant. In addition, post-reinstatement US expectancy ratings were significantly higher in preterms. However, considering early and late reinstatement there were no significant block by group interactions. Thus, return of fear responses in recall and reinstatement were not significantly different between groups. We were therefore unable to provide evidence that return of fear following extinction learning is elevated in prematurity.

### Limitations

Lack of differences between very preterm-born and term-born participants may be explained by several reasons. First, the fear conditioning paradigm used in the present study was complex. It involved the presentation of two contexts, one presented during acquisition training, the other during extinction training, recall and reinstatement. This was done to control for context-related associations, which is commonly done in the rodent literature, but rarely in the human literature^34,45,59,61^. The contexts were pictures of two different office spaces shown on a monitor to the participants. This is likely different from testing in two different chambers, which is usually done in rodent studies. The strength of the learned associations between the CS + s and the US, and the CS− and safety may have been less, because participants learned in addition the association with a complex context. Likewise, the use of two CS + s, one which got extinguished and the other not, may have hampered the strength of the CS+/US associations. The use of different contexts and two CS + s may have increased variability of outcome measures and impeded the detection of small differences between preterms and controls. Future studies are needed using less complex fear conditioning paradigms, for example using a single CS+ and a single CS−, which are presented in the same neutral context, to confirm the present results.

Of note, a semi-instructed fear conditioning paradigm was used. Semi-instructed fear conditioning is a common procedure to ensure uniform fear learning and awareness, a prerequisite to study e.g. extinction^34^. Here, the presentation of one paired CS+/US trial is often enough to learn the association. Therefore, as in the present study, stimulus × block interactions are frequently not observed^33^.

Furthermore, the use of a complex paradigm might have caused a partial suppression of the autonomic nervous system/ emotional behavior of the participants. When cognitive tasks are new and challenging, a suppression of autonomic nervous system takes place for frontal circuits to allocate neural resources according to cognitive demand^62,63^. Again, this may have hampered detection of small differences in SCR between groups. However, one may have expected group differences in the questionnaires given that preterm participants have known attention deficits.

Our findings by no means indicate that prematurity does not result in a higher incidence of clinical anxiety disorders. Indeed, as outlined above, twelve very preterm-born young adults presented with a history of emotional disorders during adolescence, and a higher percentage of very preterm-born participants (43.2%) showed mild to moderate levels of anxiety compared to controls (12.9%) based on the DASS-21. Fear conditioning abnormalities, however, may be more prominent in preterm participants presenting with more pronounced anxiety or other emotional disorders.

Furthermore, abnormalties may have been missed because of reduced autonomic responses in preterm born individuals. SCR reflects the activity of the sympathetic axis of autonomic nervous system since eccrine sweat glands are innervated by efferent fibers of the sympathetic nerves^64^. Previous studies showed an impaired autonomic nervous system functioning characterized by a limited heart rate variability in preterm-born infants as well as adolescents^65,66^. In fact, there was a tendency of preterms to show more non-zero SCRs than controls. Preterms, however, showed differential SCRs to the CS+ and CS−.

Finally, no prior power analysis was performed, and the sample size may have been too small to discover group differences. Mild differences in fear learning might become significant in a larger cohort of preterm-born young adults compared to controls.

### Conclusions

Although very preterm-born young adults were on average more anxious than their term-born peers, we did not detect significant abnormalities in the acquisition of learned differential fear responses except for mildly reduced awareness of CS-US contingencies. Likewise, extinction learning and return of fear in recall and reinstatement were not significantly altered. Small differences, however, may become more obvious using a simpler differential learning paradigm with no change in contexts and a single CS +. Abnormalities in preterms may also be more prominent in preterms who have developed more pronounced anxiety disorders. Findings need to be confirmed in future studies in larger preterm populations.

## Materials and methods

### Participants

A total of 38 preterm-born young adults and 42 age- and sex-matched term-born controls performed the experiment. Decision was made to exclude 12 participants (1 preterm participant and 11 controls) tested prior the COVID-19 pandemic and therefore without the necessary safety measures (face mask, transparent divider between examiner and participant) which likely changed the baseline level of fear. Thus, a total of 37 preterm-born participants (mean age 20.0 ± 2.8 years) and 31 controls (mean age 22.2 ± 2.3 years) were included in the final data analysis.

Preterm-born young adults were recruited from the data base of preterm infants born at the University Hospital Essen as well as via social media, flyers and posters at university and hospital. Healthy participants were recruited via social media as well as flyers and posters at university and hospital.

Inclusion criteria were as follows: (1) very preterm birth (23–≤ 32 weeks) or term birth (≥ 37 weeks), and (2) age-appropriate development at the time of the testing without special needs/education. Exclusion criteria were: (1) intra/(peri-) ventricular hemorrhage ≥ III or periventricular leukomalacia based on brain MRI or ultrasound acquired at the time of term equivalent age, (2) focal neurological disorders. For clinical description of the included participants see Table 1.

**Table 1:** Group characteristics of very preterm adults and controls.

	Very preterm (*n* = 37)	Term (*n* = 31)	*p*^a^
Clinical characteristics			
Gestational age, weeks [range]	29 + 2.8 [26 + 2–32.0]	39 + 5.7 [37.0–42.0]	** < 0.001**
Birth weight, grams [range]	1264.2 [520–2370]	3637.5 [2995–5360]	** < 0.001**
Female, *n* (%)	19 (51.4%)	16 (51.6%)	0.983
IVH < grad III, *n* (%)	1 (2.7%)	0 (0%)	1
Follow-up characteristics			
Age at assessment, years [range]	20.2 [17.8–27]	22.4 [18–29]	** < 0.001**
Education, high^b^*, n*	30	29	0.131
Parental education, high^b^, *n*	22	24	0.115
Health status^c^	5.88 [4.14–7]	5.47 [3.71–6.57]	**0.015**
IQ	97 [83–123]	108.71 [89–122]	** < 0.001**
Any therapy^d^, *n* (%)	15 (40.5%)	0 (0%)	** < 0.001**
Any psychiatric/ social-emotional disorders^e^, *n* (%)	12 (32.4%)	1 (3.2%)	**0.002**
Phobias/anxiety/depression, n (%)	5 (13.5%)	0 (0%)	
ADS/ADHS, n (%)	5 (13.5%)	0 (0%)	
Difficulties social-emotional adaptation, n (%)	2 (5.4%)	0 (0%)	
DASS-21 depression, *n* (%)	6 (16.2%)	1 (3.2%)	**0.023**
DASS-21 anxiety, *n* (%)	16 (43.2%)	4 (12.9%)	**0.032**
DASS-21 stress, *n* (%)	10 (27%)	1 (3.2%)	0.31
Developmental disorders^f^, *n* (%)	15 (40.5%)	0 (0%)	** < 0.001**

Significant values are in [bold].

Notes. Data are presented as mean (standard deviation) if not indicated otherwise.

^a^t-test or Mann–Whitney U-test and chi-square results or Fischer’s exact test for continuous and categorical data, respectively.

^b^> 10 years school.

^c^Assessment of health status based on the Life Satisfaction Questionnaire (Fragebogen zur Lebenszufriedenheit, FLZ).

^d^Having any therapies, including speech therapy, physical therapy, or occupational therapy.

^e^Psychiatric disorders including attention-deficit-(hyperactivity)-disorder, emotional disorder.

^f^Developmental disorders concerning language, gross- or fine motor functions which needed therapy in the past (speech therapy, physical therapy or occupational therapy).

SGA, small for gestational age (birth weight < 10th percentile); IVH, intraventricular haemorrhage; BPD, bronchopulmonary dysplasia; ROP, retinopathy of prematurity; IQ, intelligence quotient based on the Wechsler Adult Intelligence Scale—Third Edition.

Twelve preterm-born participants and one control had a history of emotional disorders. Five preterms developed (social) phobias and/or subclinical symptoms of anxiety or depression in their adolescence, without acute symptoms and without medication at the time of the experiment. Five preterm participants developed an attention deficit syndrome with or without hyperactivity in childhood/adolescence, without acute symptoms and without treatment at the time of the experiment. Two preterms reported difficulties in their social-emotional adaptation as a child and received occupational therapy. One control presented with a somatoform pain disorder in adolescence.

Fifteen preterm-born participants showed developmental disorders concerning language, gross- or fine motor functions which needed therapy in the past (speech therapy, physical therapy or occupational therapy). Thirtyfour very preterm-born participants and 30 controls were right-handed, two very preterm-born participants were left-handed and one very preterm-born participant and one control were ambidextrous based on the Edinburgh handedness inventory^67^. Participants were instructed to refrain from alcohol and drug intake at least 24 h prior to the experiment. All of the participants were non-smokers.

The ethics committee of the University of Duisburg-Essen approved the study (19-8890-BO). The study conforms to the principles laid down in the Declaration of Helsinki. All participants gave written informed consent. They were compensated for their participation with 80 Euros.

### Depression-Anxiety-Stress-Scale-21 (DASS-21)

The Depression-Anxiety-Stress-Scale-21 (DASS-21) questionnaire was used to assess participants’ depression, anxiety, and stress levels^68–70^. The DASS-21 is a 21-question self-report with 7 questions for each of the three subscales. On the depression subscale a score of 0–9 is within the normal range, on the anxiety subscale a score of 0–7, and on the stress subscale a score of 0–14^30^.

### Fear conditioning

**Figure 5:**
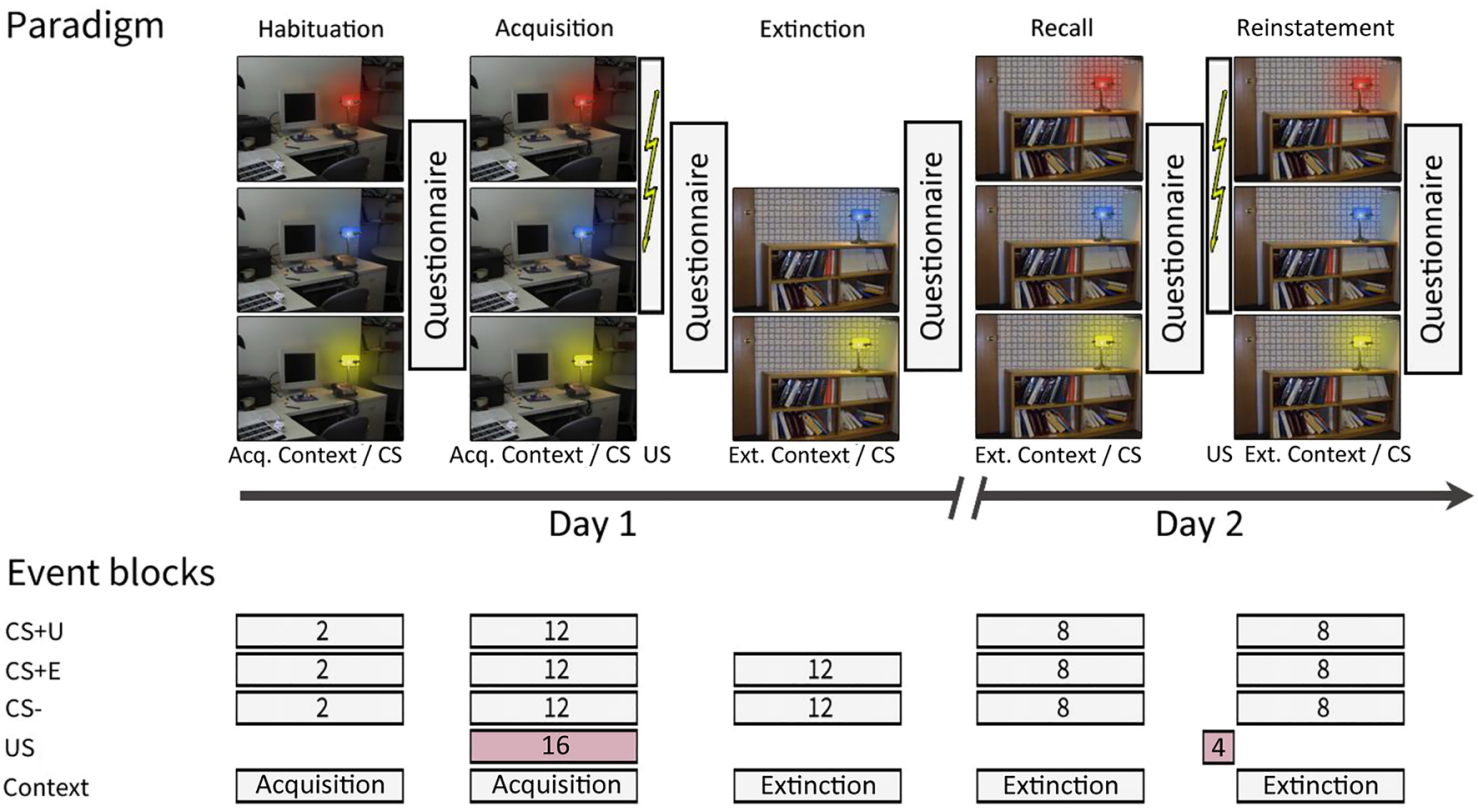
Experimental paradigm and event blocking scheme. Habituation and acquisition were performed in context A. Extinction, recall and reinstatement were performed in context B. Contexts were represented by a photography of either a desk (“office”) or a bookshelf (“library”). The CSs were represented by the same desk lamp shining either in blue, red or yellow color. For further details see text. Experimental paradigm according to Milad et al.^45^.

The experiment was performed on two consecutive days. Figure 5 displays the experimental paradigm. Habituation, acquisition training and extinction training was performed on day 1, recall and reinstatement were tested on day 2.

The experimental paradigm closely followed the paradigm introduced by Milad, Wright^59^. To emphasize conditioning to the cue and not the context, acquisition and extinction training were performed in different contexts (A and B) represented by pictures of two different office spaces. Conditioned stimuli (CS) were represented by a lamp shining either in blue, red or yellow color. The unconditioned stimulus (US) was an electric shock presented to the left calf. The same desk lamp was present in both contexts. Two CS + s and one CS− were shown in the acquisition training phase. The CS + s were both reinforced by an electric shock. Reinforcement rate was 66.6%. The CS− was never paired with the shock. In extinction training, one of the CS + s and the CS− were shown. In recall and reinstatement the extinguished (CS + E), the unextinguished (CS + U) and the CS− were shown. A CS + U was used in addition to a CS + E for direct comparison of recall of extinction (CS + E) and fear (CS + U) in early recall^45^.

The experimental protocol on day 1 consisted of three phases: “habituation” (2 CS + E only trials, 2 CS + U only trials, 2 CS− only trials, presented in acquisition context A), “acquisition training” (8 paired CS + E/US trials, 8 paired CS + U/US trials, 4 CS + E only trials, 4 CS + U only trial, 12 CS− only trials, presented in acquisition context A) and “extinction training” (12 CS + E only trials, 12 CS− only trials, presented in extinction context B). The experimental protocol on day 2 consisted of the recall phase (8 CS + E only trials, 8 CS + U only trials, 16 CS− only trials, presented in extinction context B), and the reinstatement phase (4 US-only trials in neutral background followed by 8 CS + E only trials, 8 CS + U only trials, 16 CS− only trials, presented in extinction context B) phases. A checkerboard (with four two darker and two lighter gray squares) was shown as neutral background. The different trial types in each phase were presented in pseudo-randomized order, with two restrictions: firstly, the first two trials and the very last trial of acquisition training were set to be paired CS+/US trials, and secondly, the number of events of each kind was kept identical in the first half and in the second half of the experiment.

The order of events was the same for all participants in habituation and extinction training phases. In the acquisition training, the order of CS + E and CS + U events was counterbalanced. In the recall and reinstatement phases the first event was counterbalanced between CS + E and CS + U trials.

The paradigm presentation was controlled by a computer running the software Presentation (version 20.0, Neurobehavioral System Inc., Berkeley, CA). Visual stimuli were shown on a monitor (52.2 cm × 29.4 cm) placed about 6.5 feet (ca. 1.90 m) infront of the participant. The context image was continuously displayed throughout each phase. Each trial consisted of an 8 s CS presentation. In case of reinforced trials, a 100 ms aversive US was presented co-terminating with the CS+. Intertrial intervals were randomized between 12.2 and 15.7 s. The use of context images and CS colors was pseudo-randomly counterbalanced across participants.

A short electrical stimulation was used as an aversive US. The electrical stimulation was generated by a constant current stimulator (DS7A, Digitimer Ltd., London, UK) and applied to the left calf (over the gastrocnemius muscle) via a concentric (ring-shaped) bipolar surface electrode with 6 mm conductive diameter and a central platinum pin (WASP electrode, Specialty Developments, Bexley, UK). Electrode position was marked with a permanent marker on day 1 to use the same electrode position on day 2. The 100 ms US consisted of a short train of four consecutive 500 µs current pulses (maximum output voltage: 400 V) with an interpulse interval of 33 ms. Stimulation intensity was determined immediately before start of the experiment. Stimulation current was gradually increased, and participants were asked to report on the perceived sensation intensity until an “unpleasant” but not “painful” intensity was reached. To counteract habituation to the unpleasant stimuli leading to weakening of the CRs, 20% was added to the individual thresholds^32^. The final individual current setting was kept constant for all stimulations.

A semi-instructed fear conditioning procedure was used. On both days, immediately prior to the experiment each participant read instructions on the screen, that stated that they would be shown visual stimuli and that electrical shocks would be applied during the experiment. On day 1 participants were informed, that should they perceive a pattern between stimuli, the experimenter would not change that pattern during the experiment. At the beginning of day 2, participants were informed that any pattern perceived during day 1 would stay the same on day 2. Participants confirmed that they had read and understood the instructions. Prior habituation on day 1, participants were informed that during the following phase only pictures will be shown without presentation of an electric shock. Prior acquisition training, participants were instructed that the actual experiment was about to begin and were asked to sit still and focus on the possible association between the color and the electric shock. Prior extinction training, recall and reinstatement, participants were asked to sit still and pay attention to the screen without any further instructions.

### Acquisition and evaluation of skin conductance responses

Throughout the experiment, skin conductance responses (SCRs) were measured (see Batsikadze et al.^33^ for details). SCRs were acquired using a physiological data acquisition station and appropriate hardware filters sampling at 1 kHz with a gain of 10 μS/V (MP160, BIOPAC Systems Inc., Goleta, CA). Two skin conductance electrodes were attached to the participants’ left hypothenar, approximately 2 cm apart from each other.

To eliminate high-frequency noise SCR data was low-pass filtered with a 10 Hz cutoff using a hardware filter (EDA100C-MRI module, BIOPAC Systems Inc., Goleta, CA). Semi-automated peak detection was performed using MATLAB software (Release 2019a, RRID:SCR_001622, The MathWorks Inc., Natick, MA), and SCRs were defined as the maximum trough-to-peak-amplitude of any SCR peak with a minimum amplitude of starting within a time interval from 1 to 7.999 s after CS onset^71^. SCRs were identified as local maxima with a minimum amplitude of 0.01 μS and a minimum rise time of 500 ms^72^. Trials that did not meet the criteria were scored as zero and included in the subsequent data analysis (see Supplementary data Table S2 for statistics of non-zero SCRs for individual trials).

The resulting raw SCR amplitudes were averaged in blocks and normalized through logarithmic (ln[1 + SCR]) transformation^72–74^. Two habituation trials of the same CS were combined to form single blocks. In the subsequent phases, all trials of the same CS were divided in an early block and a late block. Specifically, in the acquisition and extinction training, the averaging included the first and last six trials, while in the recall and reinstatement, the averaging included the first and last four trials. Shapiro–Wilk-test was used to test the data and the distribution of residuals for normality. Since the normality test revealed a non-normal distribution of SCRs and the residuals (*p* < 0.05), data were analysed with non-parametric statistical analysis using the PROC Mixed procedure in SAS (SAS Studio 3.8, SAS Institute Inc, Cary, NC, USA) and nparLD R package (http://www.R-project.org/). Non-parametric ANOVA-type statistics for repeated measures^75–77^ was used separately for each phase with SCR as dependent variable, stimulus (CS + E, CS + U, CS) and block (early and late phase) as within-subject factors and group (preterm, control) as between subject factor as well as their interactions. In case of significant results of non-parametric ANOVA, post-hoc comparisons were performed using least square means tests and were adjusted for multiple comparisons using the Bonferroni method. To quantify the effect sizes, we used a metric called relative treatment effects (RTE), which can range from 0 to 1. The expression *p*_*X*_ = *P (X* < *Y)* represents the the RTE with the factor level of interest (X) and a fixed reference distribution’s mean value (Y). If *p*_*X*_ < *p*_*Z*_, it implies that the data measured under condition X are generally smaller than those measured under condition Z. In contrast, *p*_X_ = *p*_Z_ indicates that there is no systematic difference between the data under conditions X and Z. An additional illustration could be that a *p*_*X*_ value of 0.25 would show the likelihood of selecting a subject randomly from the entire dataset who would score lower than a subject chosen at random from the condition X is approximately 25%.

Additionally, differential SCR were calculated as SCR to CS + s minus SCR to the CS- from the respective block^78^. Non-parametric ANOVA-type statistics for repeated measures was used separately for each phase with differential SCR as dependent variable, stimulus (CS + E minus CS− or CS + U minus CS-) and block (early and late phase) as within-subject factors and group (preterm, control) as between subject factor as well as their interactions. Results of the differential SCR analysis was not different from the previous analyses and are included in the Supplementary data (Table S6 and Fig. S7).

Finally, we examined whether male and female participants exhibited differences in fear learning. To this end, we divided the groups by sex (16 female and 15 male controls, 19 female and 18 male preterms) and run the aforementioned analyses.

### Questionnaires

Participants were asked to answer four questionnaires following each phase of the experiment (see Batsikadze et al.^33^, for details). Questions were shown on the monitor and participants gave answers using a button box with their right hand.

Participants were asked to rate their (hedonic) valence, (emotional) arousal, fear and contingency awareness on viewing images of the CS + E, CS + U and CS− on a nine-step Likert scale from "very pleasant" to "very unpleasant", "quiet and relaxed" to "very excited", “not afraid” to “very afraid” and “US not expected” to “US surely expected”, respectively. Additionally, the questionnaire following acquisition contained further questions regarding US perception and CS-US contingency: rating of the last US on a nine Likert step-scale (“not unpleasant” to “very unpleasant”), and an estimation after which time and number of US presentations, if at all, a connection between the visual stimuli and the US presentation was identified.

For conditioning and CS-US contingency awareness assessment, the valence, arousal, fear, and US expectancy ratings were analyzed using non-parametric ANOVA type statistic for repeated measures with the respective rating as a dependent variable, stimulus (CS + E, CS + U, CS−) and time (prior acquisition, post acquisition, post extinction and post recall) as within-subject factors and group (preterm, control) as between subject factor, as well as their interactions. In case of the significant results of non-parametric ANOVA, post-hoc comparisons were performed using least square means tests and were adjusted for multiple comparisons using the Bonferroni method. Additionally, we examined the effects of IQ by running analyses on questionnaire data with IQ as a covariate. Finally, we examined whether male and female participants exhibited differences in fear learning. To this end, we divided the groups by sex (16 female and 15 male controls, 19 female and 18 male preterms) and run the aforementioned analyses.

### Supplementary Information

Supplementary Information.

## Data Availability

All MATLAB and Python source code used in this paper are available uopon direct request to the corresponding author. The consent form that participants signed does not allow us to share raw data publicly, but it can be made available upon request to interested researchers through a data sharing agreement.
