# Author Correction: Fear conditioning is preserved in very preterm-born young adults despite increased anxiety levels

**DOI:** 10.1038/s41598-023-39891-z

**Published:** 2023-08-04

**Authors:** Bilge Albayrak, Lara Jablonski, Ursula Felderhoff-Mueser, Britta M. Huening, Thomas M. Ernst, Dagmar Timmann, Giorgi Batsikadze

**Affiliations:** 1 Department of Pediatrics I and C-TNBS, Pediatric and Developmental Neurology and Center for Translational Neuro- and Behavioral Sciences, University Hospital Essen, University of Duisburg-Essen, Hufelandstrasse 55, 45147 Essen, Germany; 2 Department of Neurology and C-TNBS, Essen University Hospital, University of Duisburg Essen, Hufelandstrasse 55, 45147 Essen, Germany

Correction to: *Scientific Reports* https://doi.org/10.1038/s41598-023-38391-4, published online 13 July 2023

The original version of this Article contained an error.

In the Materials and methods section, under the subheading ‘Acquisition and evaluation of skin conductance responses’,

“Throughout the experiment, skin conductance responses (SCRs) were measured (see Badsikadze et al.^33^ for details).”

now reads:

“Throughout the experiment, skin conductance responses (SCRs) were measured (see Batsikadze et al.^33^ for details).”

The original Article has been corrected.

