# Supplementary material for: Fear conditioning is preserved in very preterm-born young adults despite increased anxiety levels
Source: Sci Rep. 2023 Jul 13;13:11319. doi: 10.1038/s41598-023-38391-4 (PMC10344879; doi:10.1038/s41598-023-38391-4)
Supplement: Supplementary file 1 — Supplementary Information. [file 41598_2023_38391_MOESM1_ESM.docx]

**Fear conditioning is preserved in very preterm-born young adults despite increased anxiety levels**

Bilge Albayrak^1,*^, Lara Jablonski^1^, Ursula Felderhoff-Mueser^1^, Britta M. Huening^1^, Thomas M. Ernst^2^, Dagmar Timmann^2^, Giorgi Batsikadze^2^

^1^Dept. of Pediatrics I and C-TNBS, Essen Univ. Hosp., ^2^Dept. of Neurol. and C-TNBS, Essen Univ. Hosp., Univ. of Duisburg Essen, Hufelandstrasse 55, 45147 Essen, Germany

**Supplementary data**

**Table of contents**

**[Figures](#_Toc138097248)**[. 2](#_Toc138097248)

**[Figure S1.](#_Toc138097249)** [Skin conductance response amplitudes. Trial by trial data for control and preterm groups. 2](#_Toc138097249)

**[Figure S2.](#_Toc138097250)** [Estimates of the relative treatment effect (RTE). 3](#_Toc138097250)

**[Figure S3.](#_Toc138097251)** [Skin conductance response amplitudes. Trial by trial data for](#_Toc138097251)[non-anxious control, non-anxious preterm and anxious preterm groups. 4](#_Toc138097251)

**[Figure S4.](#_Toc138097252)** [Skin conductance response amplitudes and respective relative treatment effect (RTE) estimates. 5](#_Toc138097252)

**[Figure S5.](#_Toc138097253)** [Median subjective ratings and estimates of the respective relative treatment effect for valence, arousal](#_Toc138097253)**[,](#_Toc138097253)** [fear and US expectancy 7](#_Toc138097253)

**[Figure S6.](#_Toc138097254)** [CS-US contingency assessed after the acquisition phase. 8](#_Toc138097254)

**[Figure S7.](#_Toc138097255)** [Differential skin conductance response (CS+ − CS-) amplitudes 9](#_Toc138097255)

**Tables**[. 10](#_Toc138097256)

**[Table S1](#_Toc138097257)**[: The results of the non-parametric ANOVA-type statistics for repeated measures, analyzing skin conductance response (SCR) amplitudes, valence, arousal, fear, US expectancy ratings, and compound contingency scores, comparing the groups of preterm participants and controls. 10](#_Toc138097257)

**[Table S2:](#_Toc138097258)** [Percentages of non-zero skin conductance responses (SCRs) for individual trials in each phase of the study. 12](#_Toc138097258)

**[Table S3:](#_Toc138097259)** [Single-trial skin conductance response amplitudes. The results of the non-parametric ANOVA-type statistics for repeated measures for habituation, fear acquisition training, extinction training, recall and reinstatement. 13](#_Toc138097259)

**[Table S4:](#_Toc138097260)** [The results of the non-parametric ANOVA-type statistics for repeated measures, analyzing valence, arousal, fear, US expectancy ratings, and compound contingency scores with the participants' general IQ as a covariate. 15](#_Toc138097260)

**[Table S5:](#_Toc138097261)** [The results of the non-parametric ANOVA-type statistics for repeated measures for skin conductance response (SCR) and valence, arousal, fear and US expectancy ratings comparing the groups of anxious preterms, non-anxious preterm and non-anxious controls. 17](#_Toc138097261)

**[Table S6:](#_Toc138097262)** [Differential skin conductance response (CS+ − CS-) amplitudes. The results of the non-parametric ANOVA-type statistics for repeated measures for habituation, fear acquisition training, extinction training, recall and reinstatement. 19](#_Toc138097262)

**[Table S7:](#_Toc138097263)** [Results of the non-parametric ANOVA-type statistics for repeated measures, analyzing skin conductance response (SCR) amplitudes, valence, arousal, fear, US expectancy ratings, and compound contingency scores, comparing the groups of preterm participants and controls with the participants' sex as a covariate. 21](#_Toc138097263)

# Figures.


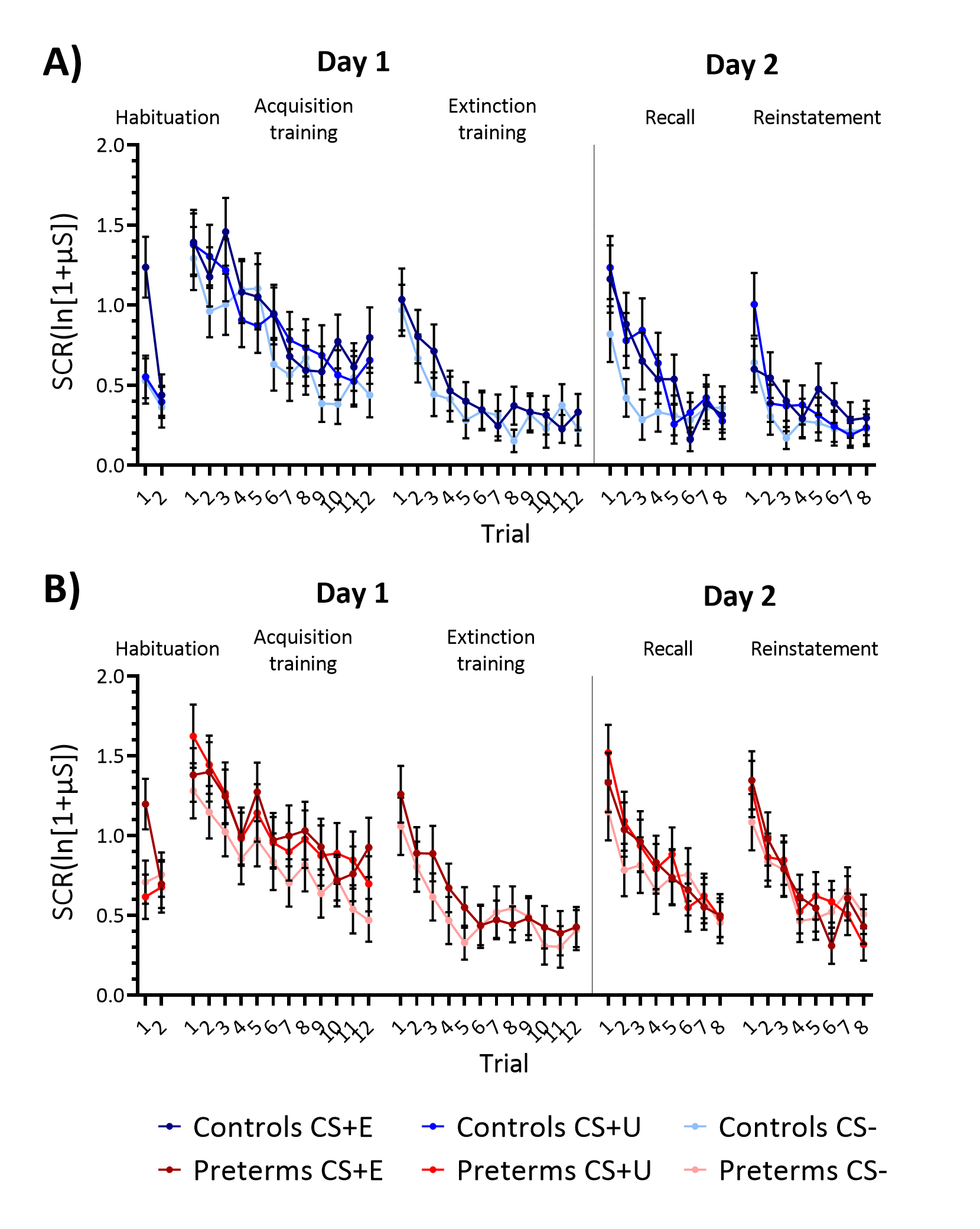


**Figure S1.** Skin conductance response amplitudes. Trial by trial data for **A)** control and **B)** preterm groups. Filled dots represent mean (log-transformed) values for individual trials for habituation, acquisition training, extinction training, recall and reinstatement phases. Error bars indicate S.E.M. Blue colors = controls, red colors = preterms. Dark colors: CS+E and CS+U, light colors: CS-.


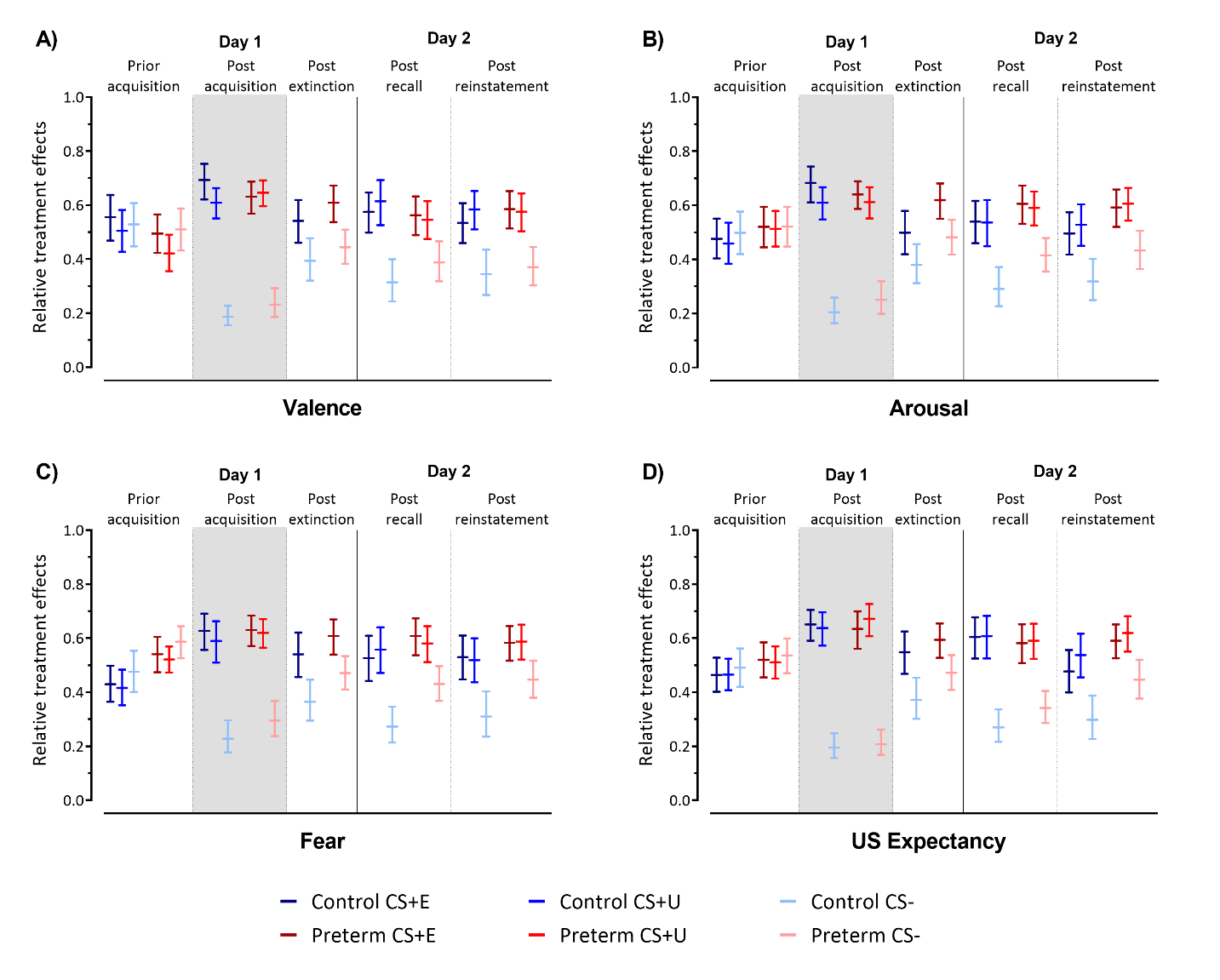


**Figure S2.** Estimates of the relative treatment effect (RTE). Median RTEs are shown comparing the subjective **A)** valence, **B)** arousal, **C)** fear and **D)** US expectancy ratings. Horizontal lines denote median values. Whiskers denote 95% confidence intervals. Blue colors = controls, red colors = preterms. Dark colors: CS+E and CS+U, light colors: CS-.


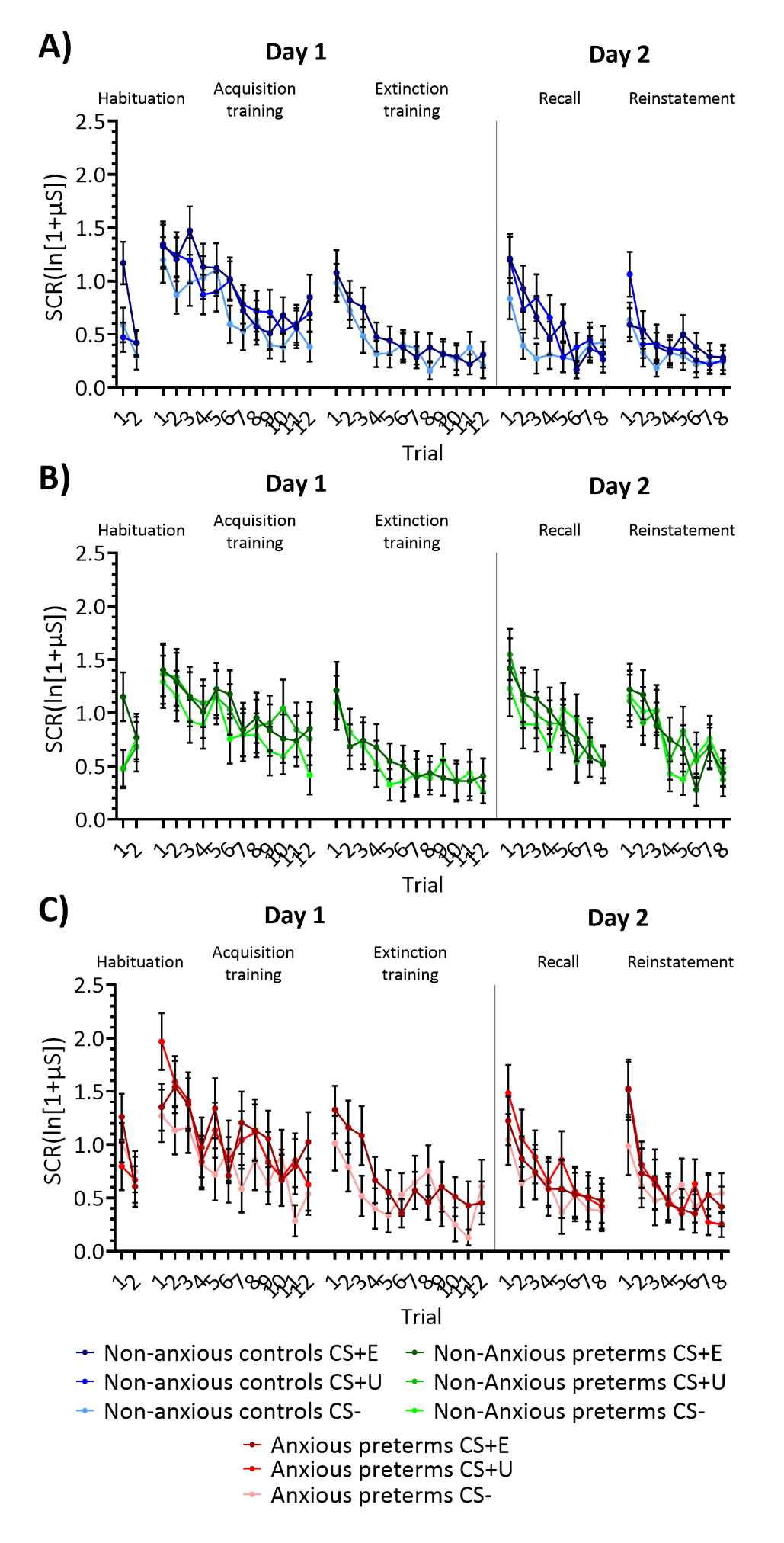


**Figure S3.** Skin conductance response amplitudes. Trial by trial data for **A)** non-anxious control, **B)** non-anxious preterm and **C)** anxious preterm groups. Filled dots represent mean (log-transformed) values for individual trials for habituation, acquisition training, extinction training, recall and reinstatement phases. Error bars indicate S.E.M. Blue colors = non-anxious controls, green colors = non-anxious preterms, red colors = anxious preterms. Dark colors: CS+E and CS+U, light colors: CS-.


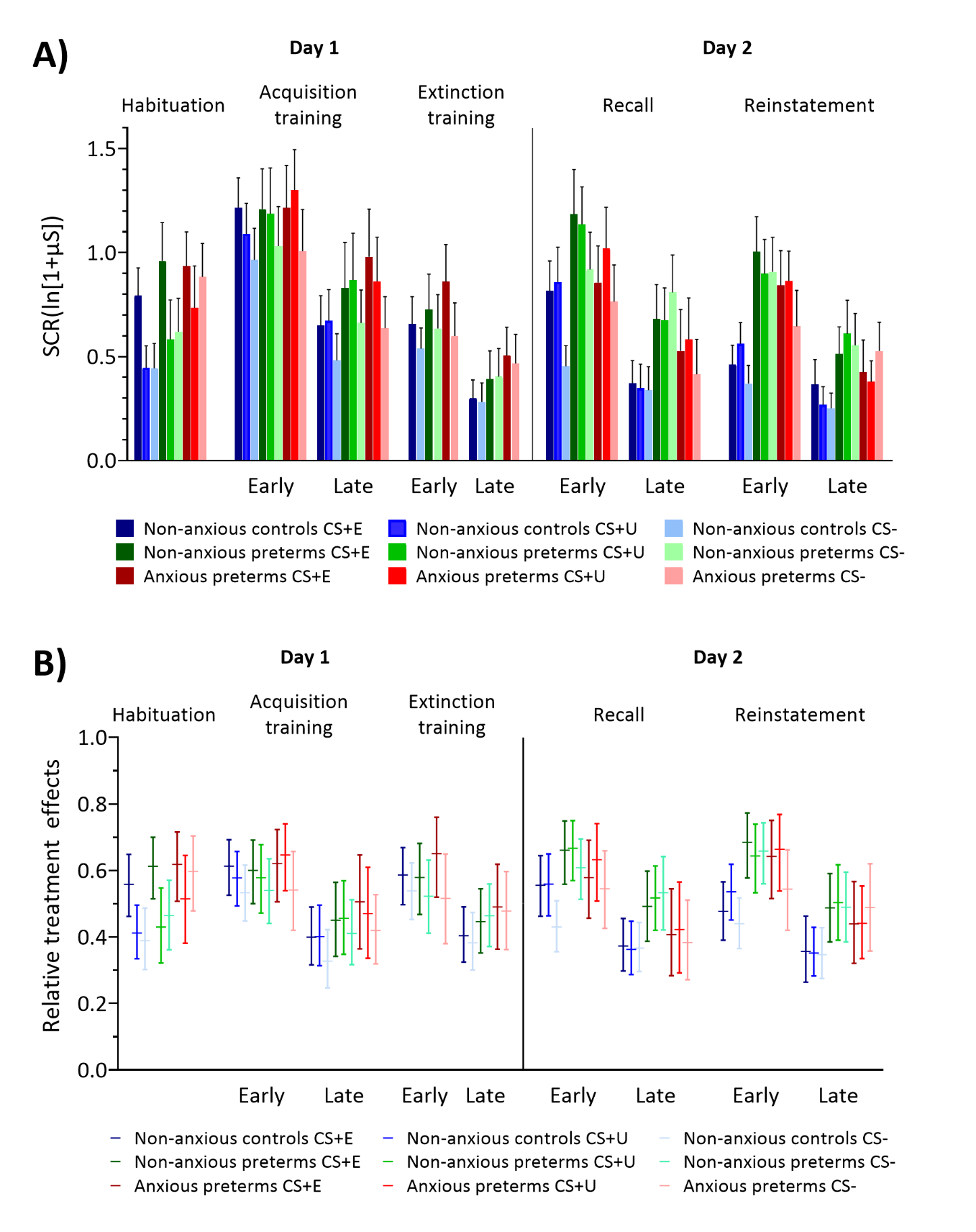


**Figure S4.** **A)** Skin conductance response amplitudes and **B)** respective relative treatment effect (RTE) estimates. **A)** Colored bars represent group mean (log-transformed) values for habituation phase and early and late blocks of acquisition training, extinction training, recall and reinstatement phases. Error bars indicate S.E.M. **B)** Horizontal lines denote median RTEs and whiskers denote 95% confidence intervals. Blue colors = non-anxious controls, green colors = non-anxious preterms, red colors = anxious preterms. Dark colors: CS+E and CS+U, light colors: CS-.


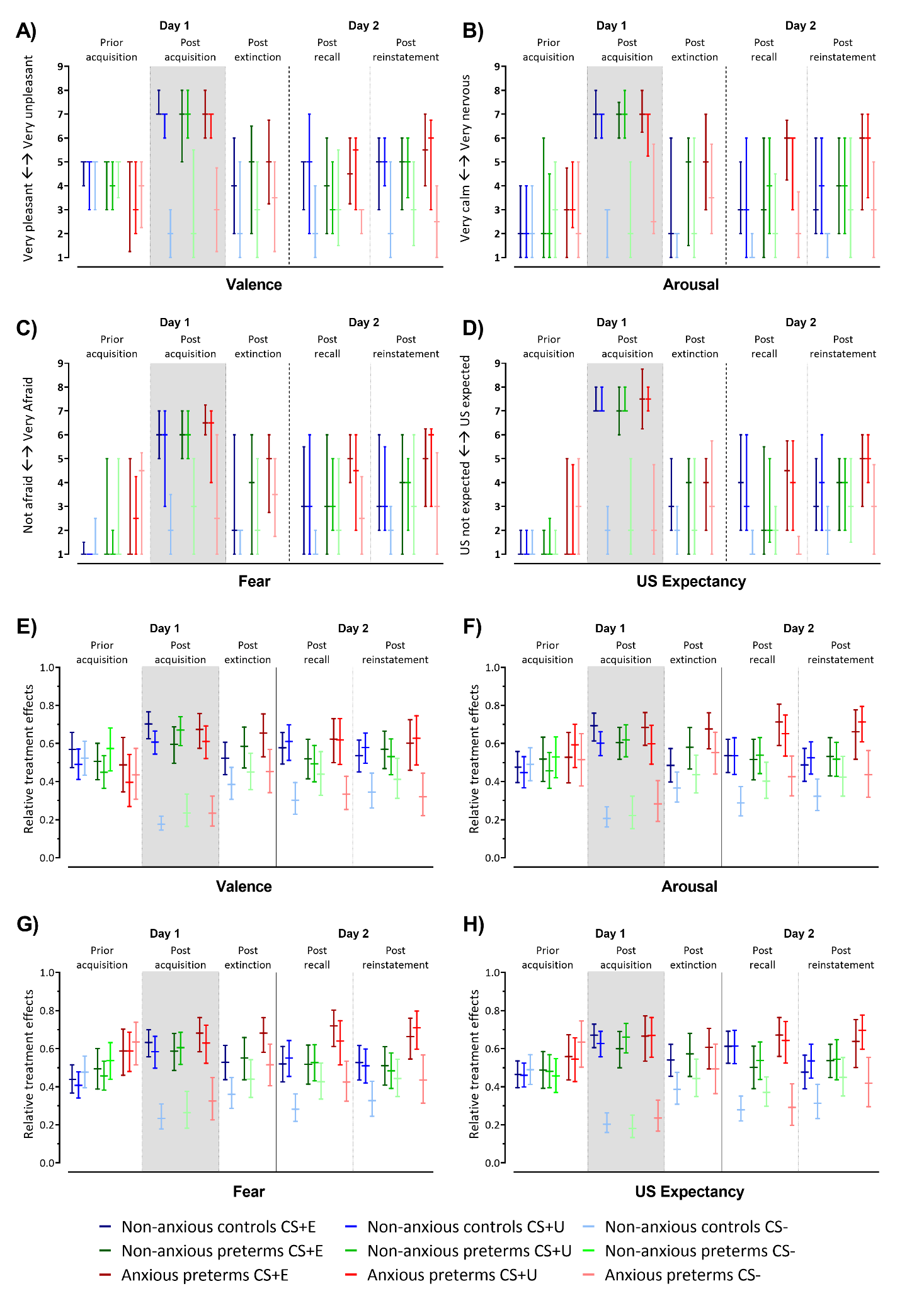


d

**Figure S5.** Median subjective ratings and estimates of the respective relative treatment effect for **A,E)** valence, **B,F)** arousal, **C,G)** fear and **D,H)** US expectancy on a Likert-scale of 1 (*“very pleasant” / “very calm” / “not afraid”, “US not* expected”, respectively) to 9 “very unpleasant” / “very nervious” / “very afraid”, “US expected”, respectively). **A-D)** Horizontal lines denote median values. Whiskers range from the first to the third quartile. **E-H)** Horizontal lines denote median values. Whiskers denote 95% confidence intervals.

Blue colors = non-anxious controls, green colors – non-anxious preterms, red colors = anxious preterms. Dark colors – CS+E and CS+U, light colors – CS–. Gray background = fear acquisition training.

**
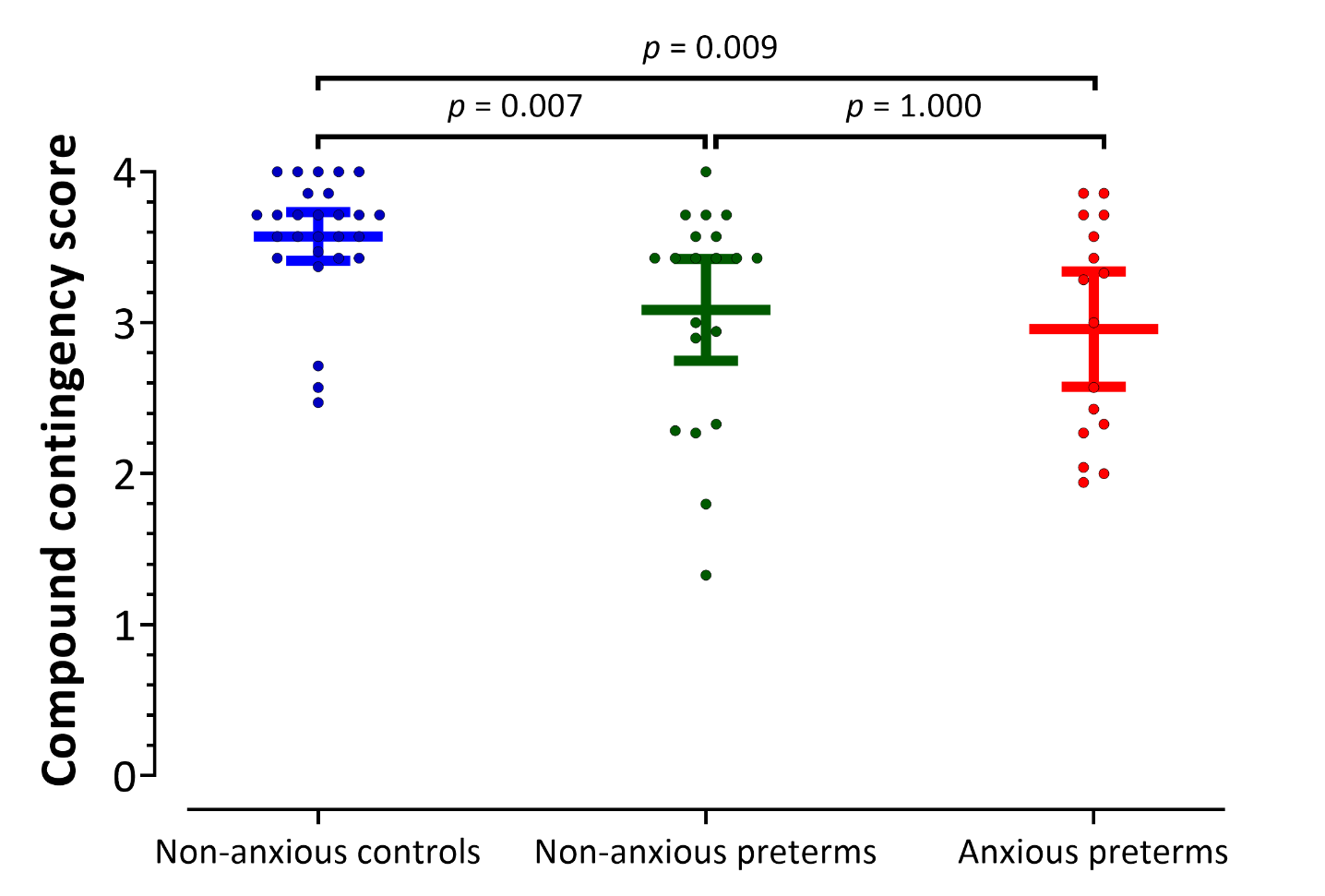
**

**Figure S6.** CS-US contingency assessed after the acquisition phase. Group mean compound contingency scores and individual data. Horizontal line represents mean value, error bars indicate 95% confidence intervals. Dots represent individual data. Blue color = non-anxious controls, green color = non-anxious preterms, red color = anxious preterms.

Post acquisition training, 1 non-anxious control (3.7 %), 3 anxious (18.8 %) and 6 non-anxious (26.6 %) preterms reported that they did not recognize a pattern between CS+ and US presentations. Regarding the CS-US contingency compound score in the participants, who recognized a pattern, non-parametric one-way ANOVA-type statistic revealed a significant group differences (*F*_(1.86.43.3)_ = 5.94, *p* = 0.006). Post-hoc pairwise comparisons showed that anxious preterms did not significantly differ from non-anxious preterms (*p* = 1.0, least square means test, corrected by Bonferroni method) and both preterm groups performed significantly below non-anxious controls (all *p* values < 0.009, least square means test, corrected by Bonferroni method).

**
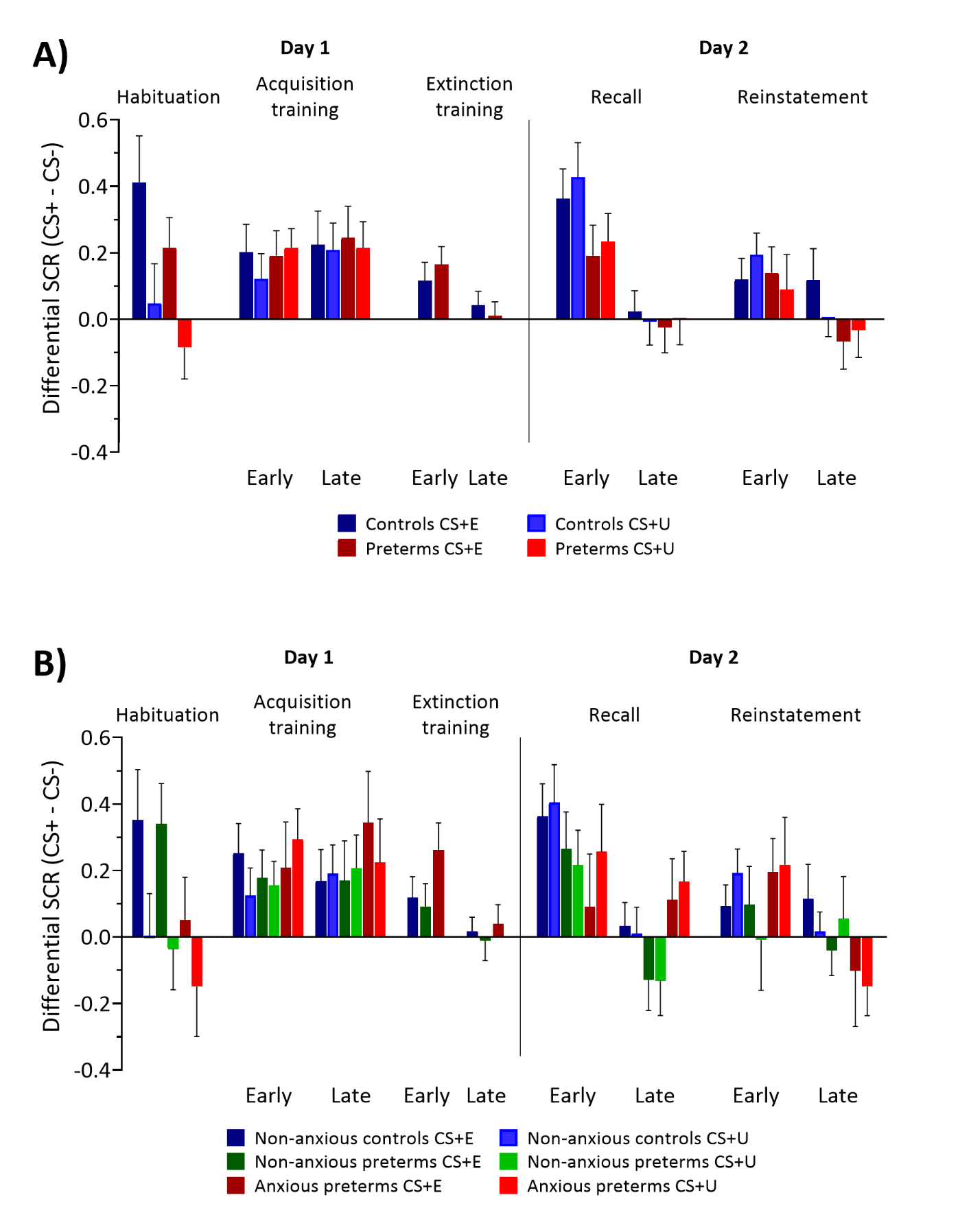
**

**Figure S7.** Differential skin conductance response (CS+ − CS-) amplitudes comparing **A)** preterm and control groups B) non-anxious preterms, anxious preterms and non-anxious controls. Colored bars represent differential mean (log-transformed) values for habituation phase and early and late blocks of acquisition training, extinction training, recall and reinstatement phases. Error bars indicate S.E.M. **A)** Blue colors = controls, red colors = preterms. **B)** Blue colors = non-anxious controls, green colors = non-anxious preterms, red colors = anxious preterms. Dark colors: CS+E, light colors: CS+U.

# Tables.

**Table S1**: The results of the non-parametric ANOVA-type statistics for repeated measures, analyzing skin conductance response (SCR) amplitudes, valence, arousal, fear, US expectancy ratings, and compound contingency scores, comparing the groups of preterm participants and controls.

| **Factor** | **Numerator Df** | **Denominator Df** | | | ***F*** | | | ***P*** | |  |
| --- | --- | --- | --- | --- | --- | --- | --- | --- | --- | --- |
| **Skin conductance responses** | | | | | | | | | |  |
| *Habituation* | | | | | | | | | |  |
| Stimulus  Group  Stimulus×Group | 1.88  1  1.88 | 103  65.9  103 | | 13.86  1.75  1.53 | | | **<.001*****  0.191  0.221 | | |  |
| *Fear acquisition training* | | | | | | | | | |  |
| Stimulus  Block  Group  Stimulus×Block  Block×Group  Stimulus×Group  Stimulus×Block×Group | 1.77  1  1  1.94  1  1.77  1.94 | 108  58.1  65.5  116  58.1  108  116 | | 16.66  90.37  0.50  0.11  2.98  0.10  0.68 | | | **<.001*****  **<.001*****  0.482  0.886  0.090  0.886  0.504 | | |  |
| *Extinction training* | | | | | | | | | |  |
| Stimulus  Block  Group  Stimulus×Block  Block×Group  Stimulus×Group  Stimulus×Block×Group | 1  1  1  1  1  1  1 | 61.5  62.6  66  55.7  62.6  61.5  55.7 | | 12.35  29.79  0.43  7.59  1.61  0.12  1.44 | | | **<.001*****  **<.001*****  0.513  **0.008***  0.210  0.730  0.236 | | |  |
| *Recall* | | | | | | | | | |  |
| Stimulus  Block  Group  Stimulus×Block  Block×Group  Stimulus×Group  Stimulus×Block×Group | 1.87  1  1  1.81  1  1.87  1.81 | 116  64.6  65.7  106  64.6  116  106 | | 6.93  56.42  2.81  8.82  0.01  1.52  1.47 | | | **0.002***  **<.001*****  0.099  **<.001*****  0.922  0.223  0.235 | | |  |
| *Reinstatement* | | | | | | | | | |  |
| Stimulus  Block  Group  Stimulus×Block  Block×Group  Stimulus×Group  Stimulus×Block×Group | 2  1  1  1.88  1  2  1.88 | 118  63  65.9  104  63  118  104 | | 1.94  38.28  6.96  3.89  0.67  0.42  1.26 | | | 0.148  **<.001*****  **0.010***  **0.023***  0.414  0.660  0.287 | | |  |
| **Valence** | | | | | | | | | |  |
| Stimulus  Time  Group  Stimulus×Time  Time×Group  Stimulus×Group  Stimulus×Time×Group | 1.55  3.41  1  5.09  3.41  1.55  5.09 | 98  211  65.8  306  211  98  306 | | 67.87  24.28  0.00  37.34  0.95  0.95  1.07 | | | **<.001*****  **<.001*****  0.947  **<.001*****  0.428  0.369  0.378 | | |  |
| **Arousal** | | | | | | | | | |  |
| Stimulus  Time  Group  Stimulus×Time  Time×Group  Stimulus×Group  Stimulus×Time×Group | 1.49  3.38  1  4.77  3.38  1.49  4.77 | 90.2  218  64.9  286  218  90.2  286 | | 78.72  40.47  2.62  29.77  1.19  0.34  0.51 | | | **<.001*****  **<.001*****  0.110  **<.001*****  0.314  0.651  0.761 | | |  |
| **Fear** | | | | | | | | | |  |
| Stimulus  Time  Group  Stimulus×Time  Time×Group  Stimulus×Group  Stimulus×Time×Group | 1.41  3.21  1  4.77  3.21  1.41  4.77 | 80.8  204  65.8  272  204  80.8  272 | | 49.53  45.69  3.92  26.95  0.72  1.19  0.74 | | | **<.001*****  **<.001*****  **0.048***  **<.001*****  0.552  0.293  0.585 | | |  |
| **US expectancy** | | | | | | | | | |  |
| Stimulus  Time  Group  Stimulus×Time  Time×Group  Stimulus×Group  Stimulus×Time×Group | 1.68  3.28  1  5.04  3.28  1.68  5.04 | 94.9  208  65.9  309  208  94.9  309 | | 90.22  72.65  1.91  42.08  1.33  0.75  0.62 | | | **<.001*****  **<.001*****  0.171  **<.001*****  0.263  0.454  0.686 | | |  |
| **Compound contingency score** | | | | | | | | |  |  |
| Group | 1 | 65 | 13.54 | | | **<.001***** | | | | |

***** Significant results at *p* < 0.05.

******* Significant results at *p* < 0.001.

## **Table S2:** Percentages of non-zero skin conductance responses (SCRs) for individual trials in each phase of the study.

| **Phase** | **Group** | **%** | **Results of one-way non-parametric ANOVA-type statistic** |
| --- | --- | --- | --- |
| **Two groups (all preterms vs all controls)** | | | |
| *Habituation* | Preterms | 55 ± 33.3 | *F*_(1,64.6)_ = 1.49, *p* = 0.226 |
|  | Controls | 44.4 ± 32.6 |  |
| *Acquisition training* | Preterms | 61 ± 31 | *F*_(1,64.2)_ = 0.44, *p* = 0.511 |
|  | Controls | 53.7 ± 29.1 |  |
| *Extinction training* | Preterms | 43 ± 32.1 | *F*_(1,65.6)_ = 0.49, *p* = 0.487 |
|  | Controls | 36 ± 28.8 |  |
| *Recall* | Preterms | 52.8 ± 31.8 | *F*_(1,64.9)_ = 1.36, *p* = 0.247 |
|  | Controls | 42.1 ± 30.1 |  |
| *Reinstatement* | Preterms | 53 ± 28.4 | *F*_(1,64.3)_ = 5.17, *p* = **0.026*** |
|  | Controls | 35.4 ± 25.8 |  |
| **Three groups (anxious preterms vs non-anxious preterms vs non-anxious controls)** | | | |
| *Habituation* | Non-anxious preterms | 49 ± 31.4 | *F*_(1.92,47.9)_ = 1.92, *p* = 0.159 |
|  | Anxious preterms | 62.5 ± 34.7 |  |
|  | Non-anxious controls | 44.8 ± 31.7 |  |
| *Acquisition training* | Non-anxious preterms | 59.5 ± 31.7 | *F*_(1.95,50.3)_ = 0.23, *p* = 0.791 |
|  | Anxious preterms | 61.9 ± 31 |  |
|  | Non-anxious controls | 55.6 ± 30.7 |  |
| *Extinction training* | Non-anxious preterms | 40.5 ± 32.6 | *F*_(1.94,50.4)_ = 0.48, *p* = 0.614 |
|  | Anxious preterms | 47.5 ± 31.1 |  |
|  | Non-anxious controls | 38.1 ± 29.4 |  |
| *Recall* | Non-anxious preterms | 58.6 ± 30.7 | *F*_(1.94,49.1)_ = 1.63, *p* = 0.207 |
|  | Anxious preterms | 45 ± 32.5 |  |
|  | Non-anxious controls | 41.9 ± 31.5 |  |
| *Reinstatement* | Non-anxious preterms | 55.7 ± 30.6 | *F*_(1.97,52.6)_ = 2.29, *p* = 0.112 |
|  | Anxious preterms | 51.3 ± 27.3 |  |
|  | Non-anxious controls | 37 ± 29.6 |  |

***** Significant results at *p* < 0.05.

## **Table S3:** Single-trial skin conductance response amplitudes. The results of the non-parametric ANOVA-type statistics for repeated measures for habituation, fear acquisition training, extinction training, recall and reinstatement.

The observed Stimulus effect and Stimulus×Trial interaction in habituation may be attributed to the fact that CS+E trials were consistently presented first, and both groups exhibited significantly higher SCR amplitudes in response to these trials compared to the rest. Post hoc pairwise comparisons revealed significant differences between the first CS+E trials and the first CS+U and CS- trials (all *p* values < 0.001, least squares means test), indicating that the first CS+E trial elicited a significantly higher SCRs than the first CS+U and CS- trials. However, these differences disappeared in the second habituation trial (all *p* values = 1.0, least squares means test), indicating that this effect was limited to the initial habituation phase.

| **Factor** | **Numerator Df** | **Denominator Df** | **F** | **P** |
| --- | --- | --- | --- | --- |
| **Two groups (all preterms *vs* all controls)** | | | | |
| *Habituation* | | | | |
| Stimulus  Trial  Group  Stimulus×Trial  Trial×Group  Stimulus×Group  Stimulus×Trial×Group | 1.91  1  1  1.99  1  1.91  1.99 | 107  65.1  66  120  65.1  107  120 | 10.65  14.18  1.80  9.56  2.44  1.23  0.06 | **<.001*****  **<.001*****  0.184  **<.001*****  0.123  0.294  0.943 |
| *Fear acquisition training* | | | | |
| Stimulus  Trial  Group  Stimulus×Trial  Trial×Group  Stimulus×Group  Stimulus×Trial×Group | 1.82  5.4  1  13.7  5.4  1.82  13.7 | 120  356  66  903  356  120  903 | 15.36  22.42  0.63  0.70  0.94  0.14  0.97 | **<.001*****  **<.001*****  0.431  0.773  0.459  0.850  0.479 |
| *Extinction training* | | | | |
| Stimulus  Trial  Group  Stimulus×Trial  Trial×Group  Stimulus×Group  Stimulus×Trial×Group | 1  7.12  1  8.22  7.12  1  8.22 | 66  470  66  543  470  66  543 | 27.04  16.20  0.84  1.07  0.65  0.02  0.64 | **<.001*****  **<.001*****  0.361  0.383  0.716  0.882  0.753 |
| *Recall* | | | | |
| Stimulus  Trial  Group  Stimulus×Trial  Trial×Group  Stimulus×Group  Stimulus×Trial×Group | 1.87  3.18  1  9.9  3.18  1.87  9.9 | 123  210  66  653  210  123  653 | 5.87  17.51  2.73  1.90  0.50  0.87  0.74 | **0.005***  **<.001*****  0.104  **0.042***  0.691  0.416  0.683 |
| *Reinstatement* | | | | |
| Stimulus  Trial  Group  Stimulus×Trial  Trial×Group  Stimulus×Group  Stimulus×Trial×Group | 2  4.59  1  10.9  4.59  2  10.9 | 132  303  66  721  303  132  721 | 1.28  12.73  7.08  0.81  1.02  0.13  0.93 | 0.280  **<.001*****  **0.010***  0.630  0.405  0.878  0.510 |
| **Three groups (anxious preterms *vs* non-anxious preterms *vs* non-anxious controls)** | | | | |
| *Habituation* | | | | |
| Stimulus  Trial  Group  Stimulus×Trial  Trial×Group  Stimulus×Group  Stimulus×Trial×Group | 1.9  1  1.96  1.99  1.96  3.72  3.9 | 116  61  61  122  61  116  122 | 8.77  10.50  1.51  8.56  3.53  0.99  0.39 | **<0.001*****  **0.002***  0.229  **<0.001*****  **0.036***  0.411  0.811 |
| *Fear acquisition training* | | | | |
| Stimulus  Trial  Group  Stimulus×Trial  Trial×Group  Stimulus×Group  Stimulus×Trial×Group | 1.84  5.41  1.96  13.3  10.6  3.6  26 | 112  330  61  811  330  112  811 | 13.64  18.99  0.28  0.63  1.09  0.33  1.29 | **<0.001*****  **<0.001*****  0.756  0.834  0.366  0.836  0.153 |
| *Extinction training* | | | | |
| Stimulus  Trial  Group  Stimulus×Trial  Trial×Group  Stimulus×Group  Stimulus×Trial×Group | 1  6.85  1.96  7.87  13.4  1.96  15.4 | 61  418  61  480  418  61  480 | 27.30  14.65  0.49  1.06  0.65  2.51  1.30 | **<.001*****  **<.001*****  0.610  0.387  0.813  0.091  0.197 |
| *Recall* | | | | |
| Stimulus  Trial  Group  Stimulus×Trial  Trial×Group  Stimulus×Group  Stimulus×Trial×Group | 1.88  3.03  1.96  9.94  5.93  3.67  19.4 | 114  185  61  606  185  114  606 | 4.51  16.19  1.73  1.33  0.41  0.68  0.87 | **0.015***  **<.001*****  0.187  0.208  0.867  0.596  0.622 |
| *Reinstatement* | | | | |
| Stimulus  Trial  Group  Stimulus×Trial  Trial×Group  Stimulus×Group  Stimulus×Trial×Group | 1.99  4.76  1.96  10.6  9.32  3.88  20.6 | 121  291  61  644  291  121  644 | 0.78  14.35  2.91  0.87  1.63  0.05  1.00 | 0.462  **<.001*****  0.063  0.568  0.103  0.995  0.465 |

***** Significant results at *p* < 0.05.

******* Significant results at *p* < 0.001.

## **Table S4:** The results of the non-parametric ANOVA-type statistics for repeated measures, analyzing valence, arousal, fear, US expectancy ratings, and compound contingency scores with the participants' general IQ as a covariate.

| **Factor** | **Numerator Df** | | | **Denominator Df** | | | | | | | **F** | | | | **P** | |
| --- | --- | --- | --- | --- | --- | --- | --- | --- | --- | --- | --- | --- | --- | --- | --- | --- |
| **Two groups (all preterms *vs* all controls)** | | | | | | | | | | | | | | | | |
| **Valence** | | | | | | | | | | | | | | | | |
| Stimulus  Time  Group  Stimulus×Time  Time×Group  Stimulus×Group  Stimulus×Time×Group  IQ | | 1.55  3.42  1  5.12  3.42  1.55  5.12  1 | | | 97.9  210  63.9  306  210  97.9  306  67.9 | | | | 66.44  24.88  0.09  37.94  0.86  0.94  1.00  0.24 | | | | | **<.001*****  **<.001*****  0.761  **<.001*****  0.473  0.373  0.420  0.623 | | |
| **Arousal** | | | | | | | | | | | | | | | | |
| Stimulus  Time  Group  Stimulus×Time  Time×Group  Stimulus×Group  Stimulus×Time×Group  IQ | | 1.51  3.37  1  4.75  3.37  1.51  4.75  1 | | | 90.6  215  64.5  284  215  90.6  284  59.6 | | | | 75.97  39.94  2.37  30.06  1.07  0.46  0.43  1.36 | | | | | **<.001*****  **<.001*****  0.129  **<.001*****  0.367  0.578  0.815  0.248 | | |
| **Fear** | | | | | | | | | | | | | | | | |
| Stimulus  Time  Group  Stimulus×Time  Time×Group  Stimulus×Group  Stimulus×Time×Group  IQ | | 1.42  3.22  1  4.78  3.22  1.42  4.78  1 | | | 82  203  63.7  274  203  82  274  62.8 | | | | 48.44  44.69  4.08  26.88  0.73  1.16  0.71  0.16 | | | | | **<.001*****  **<.001*****  **0.048***  **<.001*****  0.547  0.304  0.608  0.688 | | |
| **US expectancy** | | | | | | | | | | | | | | | | |
| Stimulus  Time  Group  Stimulus×Time  Time×Group  Stimulus×Group  Stimulus×Time×Group  IQ | | 1.69  3.31  1  5.01  3.31  1.69  5.01  1 | | | 95.1  206  63.5  301  206  95.1  301  68.3 | | | | 87.34  70.50  1.63  41.07  1.35  0.87  0.66  0.78 | | | | | **<.001*****  **<.001*****  0.206  **<.001*****  0.257  0.405  0.658  0.381 | | |
| **Compound contingency score** | | | | | | | | | | | | | | | |  |
| Group  IQ | | 1  1 | | | 64  63.8 | | 15.02  2.39 | | | | | **<.001*****  0.127 | | | |  |
| **Three groups (anxious preterms *vs* non-anxious preterms *vs* non-anxious controls)** | | | | | | | | | | | | | | | | |
| **Valence** | | | | | | | | | | | | | | | | |
| Stimulus  Time  Group  Stimulus×Time  Time×Group  Stimulus×Group  Stimulus×Time×Group  IQ | | 1.66  3.41  1.87  5.29  6.19  3.2  9.08  1 | | | 83.4  154  44  206  154  83.4  206  53.9 | | | | | 63.62  23.36  0.03  32.15  0.91  1.76  1.19  4.68 | | | | | **<.001*****  **<.001*****  0.962  **<.001*****  0.495  0.157  0.301  **0.035*** | |
| **Arousal** | | | | | | | | | | | | | | | | |
| Stimulus  Time  Group  Stimulus×Time  Time×Group  Stimulus×Group  Stimulus×Time ×Group  IQ | | 1.58  3.2  1.93  5.14  5.76  3.04  8.58  1 | | | 78.2  135  51  189  135  78.2  189  61.4 | | | | | 74.47  33.07  2.78  24.64  0.82  0.68  1.42  0.03 | | | | | **<.001*****  **<.001*****  0.074  **<.001*****  0.550  0.566  0.187  0.873 | |
| **Fear** | | | | | | | | | | | | | | | | |
| Stimulus  Time  Group  Stimulus×Time  Time×Group  Stimulus×Group  Stimulus×Time ×Group  IQ | | 1.52  3.09  1.88  5.12  5.53  2.9  9.08  1 | | | 74.3  132  46.5  227  132  74.3  227  63.7 | | | | | 47.46  38.68  3.86  26.07  0.44  1.39  1.48  0.35 | | | | | **<.001*****  **<.001*****  **0.031***  **<.001*****  0.841  0.253  0.157  0.558 | |
| **US expectancy** | | | | | | | | | | | | | | | | |
| Stimulus  Time  Group  Stimulus×Time  Time×Group  Stimulus×Group  Stimulus×Time×Group  IQ | | 1.89  3.32  1.91  4.85  5.98  3.47  8.13  1 | | | 84  141  48.2  173  141  84  173  83.2 | | | | | 78.53  58.09  2.18  34.96  0.76  0.40  1.91  0.49 | | | | | **<.001*****  **<.001*****  0.126  **<.001*****  0.606  0.784  0.060  0.486 | |
| **Compound contingency score** | | | | | | | | | | | | | | | | |
| Group  IQ | | | 1.92  1 | | | 45.7  57.8 | | 6.67  1.86 | | | | | **0.003***  0.178 | | | |

***** Significant results at *p* < 0.05.

******* Significant results at *p* < 0.001.

## **Table S5:** The results of the non-parametric ANOVA-type statistics for repeated measures for skin conductance response (SCR) and valence, arousal, fear and US expectancy ratings comparing the groups of anxious preterms, non-anxious preterm and non-anxious controls.

| **Factor** | **Numerator Df** | **Denominator Df** | **F** | **P** |
| --- | --- | --- | --- | --- |
| **Skin conductance responses** | | | | |
| *Habituation* | | | | |
| Stimulus  Group  Stimulus×Group | 1.94  1.91  3.72 | 107  47.5  107 | 12.74  1.34  1.33 | **<.001*****  0.271  0.265 |
| *Fear acquisition training* | | | | |
| Stimulus  Trial  Group  Stimulus×Block  Block×Group  Stimulus×Group  Stimulus×Block×Group | 1.82  1  1.94  2  2  3.57  3.44 | 94.4  59.1  50.4  73.8  59.1  94.4  73.8 | 15.51  81.62  0.27  0.04  1.47  0.41  0.65 | **<.001*****  **<.001*****  0.760  0.963  0.237  0.780  0.609 |
| *Extinction training* | | | | |
| Stimulus  Trial  Group  Stimulus×Block  Block×Group  Stimulus×Group  Stimulus×Block×Group | 1  1  1.92  1  1.84  1.91  1.89 | 48.1  42.5  48.1  46.4  42.5  48.1  46.4 | 13.89  23.72  0.23  10.37  0.92  2.03  1.47 | **<.001*****  **<.001*****  0.785  **0.002***  0.398  0.145  0.241 |
| *Recall* | | | | |
| Stimulus  Trial  Group  Stimulus×Block  Block×Group  Stimulus×Group  Stimulus×Block×Group | 1.84  1  1.93  1.73  1.94  3.53  3.13 | 90.3  49.1  48.1  72.2  49.1  90.3  72.2 | 5.62  52.10  1.63  4.91  0.48  0.93  0.76 | **0.006***  **<.001*****  0.208  **0.013***  0.614  0.444  0.524 |
| *Reinstatement* | | | | |
| Stimulus  Trial  Group  Stimulus×Block  Block×Group  Stimulus×Group  Stimulus×Block×Group | 1.96  1  1.93  1.92  1.97  3.47  3.6 | 79.9  52.6  48.7  99.3  52.6  79.9  99.3 | 1.28  40.32  2.57  3.83  0.20  0.36  2.19 | 0.284  **<.001*****  0.088  **0.027***  0.815  0.808  0.082 |
| **Valence** | | | | |
| Stimulus  Time  Group  Stimulus×Time  Time×Group  Stimulus×Group  Stimulus×Time×Group | 1.63  3.39  1.94  5.25  6.27  3.16  9.13 | 82.8  161  49.9  210  161  82.8  210 | 64.35  22.27  0.05  32.57  0.97  1.76  1.32 | **<.001*****  **<.001*****  0.945  **<.001*****  0.451  0.158  0.228 |
| **Arousal** | | | | |
| Stimulus  Time  Group  Stimulus×Time  Time×Group  Stimulus×Group  Stimulus×Time×Group | 1.58  3.23  1.94  5.1  5.94  3.06  8.69 | 80.7  143  53.5  196  143  80.7  196 | 70.36  32.58  2.92  23.48  0.90  0.62  1.36 | **<.001*****  **<.001*****  0.064  **<.001*****  0.493  0.610  0.210 |
| **Fear** | | | | |
| Stimulus  Time  Group  Stimulus×Time  Time×Group  Stimulus×Group  Stimulus×Time×Group | 1.53  3.12  1.91  5.16  5.7  2.92  9.15 | 75.2  140  50.7  229  140  75.2  229 | 43.91  37.02  3.59  24.66  0.44  1.15  1.38 | **<.001*****  **<.001*****  **0.037***  **<.001*****  0.843  0.333  0.198 |
| **US expectancy** | | | | |
| Stimulus  Time  Group  Stimulus×Time  Time×Group  Stimulus×Group  Stimulus×Time×Group | 1.88  3.38  1.95  4.87  6.07  3.49  8.36 | 85.5  142  52.1  182  142  85.5  182 | 72.98  58.79  1.83  33.61  0.76  0.26  1.85 | **<.001*****  **<.001*****  0.171  **<.001*****  0.602  0.878  0.067 |

***** Significant results at *p* < 0.05.

******* Significant results at *p* < 0.001.

## **Table S6:** Differential skin conductance response (CS+ − CS-) amplitudes. The results of the non-parametric ANOVA-type statistics for repeated measures for habituation, fear acquisition training, extinction training, recall and reinstatement.

The observed Stimulus effect in habituation can be attributed to the fact that CS+E trials were consistently presented first, and both groups exhibited significantly higher SCR amplitudes in response to these trials compared to the rest (see also **Table S3**).

| **Factor** | **Numerator Df** | | **Denominator Df** | | | **F** | | **P** |
| --- | --- | --- | --- | --- | --- | --- | --- | --- |
| **Two groups (all preterms *vs* all controls)** | | | | | | | | |
| *Habituation* | | | | | | | | |
| Stimulus  Group  Stimulus×Group | | 1  1  1 | | 64.7  59.5  64.7 | 18.10  1.73  0.48 | | **<.001*****  0.194  0.491 | |
| *Fear acquisition training* | | | | | | | | |
| Stimulus  Block  Group  Stimulus×Block  Block×Group  Stimulus×Group  Stimulus×Block×Group | | 1  1  1  1  1  1  1 | | 66  58.5  62.8  64.3  58.5  66  64.3 | 0.02  0.11  0.25  0.85  0.05  0.13  0.58 | | 0.890  0.739  0.618  0.360  0.822  0.721  0.450 | |
| *Extinction training* | | | | | | | | |
| Block  Group  Block×Group | | 1  1  1 | | 63.6  64.4  63.6 | 5.95  0.09  0.14 | | **0.018***  0.760  0.710 | |
| *Recall* | | | | | | | | |
| Stimulus  Block  Group  Stimulus×Block  Block×Group  Stimulus×Group  Stimulus×Block×Group | | 1  1  1  1  1  1  1 | | 60.4  66  63.6  65.2  66  60.4  65.2 | 1.48  17.04  2.16  0.26  2.30  0.07  2.17 | | 0.229  **<.001***  0.147  0.613  0.134  0.791  0.146 | |
| *Reinstatement* | | | | | | | | |
| Stimulus  Block  Group  Stimulus×Block  Block×Group  Stimulus×Group  Stimulus×Block×Group | | 1  1  1  1  1  1  1 | | 66  66  64.7  65.8  66  66  65.8 | 0.41  8.16  1.21  0.91  0.30  0.10  2.48 | | 0.527  **0.006***  0.275  0.344  0.585  0.757  0.120 | |
| **Three groups (anxious preterms *vs* non-anxious preterms *vs* non-anxious controls)** | | | | | | | | |
| *Habituation* | | | | | | | | |
| Stimulus  Group  Stimulus×Group | | 1  1.99  1.91 | | 46.6  55.5  46.6 | 13.28  0.95  1.41 | | **<.001*****  0.391  0.255 | |
| *Fear acquisition training* | | | | | | | | |
| Stimulus  Block  Group  Stimulus×Block  Block×Group  Stimulus×Group  Stimulus×Block×Group | | 1  1  1.98  1  1.97  1.9  1.61 | | 47  52.1  55.6  31.5  52.1  47  31.5 | 0.05  0.55  0.42  0.46  0.05  0.17  0.74 | | 0.816  0.463  0.658  0.503  0.948  0.834  0.456 | |
| *Extinction training* | | | | | | | | |
| Block  Group  Block×Group | | 1  1.92  1.98 | | 56.2  47.4  56.2 | 6.70  2.20  0.52 | | **0.012***  0.124  0.598 | |
| *Recall* | | | | | | | | |
| Stimulus  Block  Group  Stimulus×Block  Block×Group  Stimulus×Group  Stimulus×Block×Group | | 1  1  1  1.95  1.8  1.91  1.72 | | 41.8  50.8  36.2  49.9  41.8  50.8  36.2 | 1.82  9.42  0.05  0.96  1.32  1.58  0.72 | | 0.184  **0.003***  0.828  0.387  0.277  0.216  0.472 | |
| *Reinstatement* | | | | | | | | |
| Stimulus  Block  Group  Stimulus×Block  Block×Group  Stimulus×Group  Stimulus×Block×Group | | 1  1  1.69  1  1.69  1.93  1.9 | | 49.8  44.3  34.8  46.6  44.3  49.8  46.6 | 0.16  6.91  0.46  0.20  1.07  0.18  2.35 | | 0.690  **0.012***  0.602  0.660  0.342  0.824  0.110 | |

***** Significant results at *p* < 0.05.

******* Significant results at *p* < 0.001.

## **Table S7:** The results of the non-parametric ANOVA-type statistics for repeated measures, analyzing skin conductance response (SCR) amplitudes, valence, arousal, fear, US expectancy ratings, and compound contingency scores, comparing the groups of preterm participants and controls with the participants' sex as a covariate.

| **Factor** | **Numerator Df** | | **Denominator Df** | | | **F** | | **P** |  |
| --- | --- | --- | --- | --- | --- | --- | --- | --- | --- |
| **Skin conductance responses** | | | | | | | | | |
| *Habituation* | | | | | | | | |  |
| Stimulus  Group  Stimulus×Group  Sex | | 1.88  1  1.88  1 | | 103  64.9  103  63.5 | 13.86  1.73  1.53  0.30 | | **<.001*****  0.193  0.221  0.585 | |  |
| *Fear acquisition training* | | | | | | | | |  |
| Stimulus  Block  Group  Stimulus×Block  Block×Group  Stimulus×Group  Stimulus×Block×Group  Sex | | 1.77  1  1  1.94  1  1.77  1.94  1 | | 108  58.1  62.4  116  58.1  108  116  62.8 | 16.66  90.37  0.49  0.11  2.98  0.10  0.68  0.00 | | **<.001*****  **<.001*****  0.485  0.886  0.090  0.886  0.504  0.997 | |  |
| *Extinction training* | | | | | | | | |  |
| Stimulus  Block  Group  Stimulus×Block  Block×Group  Stimulus×Group  Stimulus×Block×Group  Sex | | 1  1  1  1  1  1  1  1 | | 61.5  62.6  64.9  55.7  62.6  61.5  55.7  63.7 | 12.35  29.79  0.43  7.59  1.61  0.12  1.44  0.03 | | **0.001***  **<.001*****  0.516  **0.008***  0.210  0.730  0.236  0.858 | |  |
| *Recall* | | | | | | | | |  |
| Stimulus  Block  Group  Stimulus×Block  Block×Group  Stimulus×Group  Stimulus×Block×Group  Sex | | 1.87  1  1  1.81  1  1.87  1.81  1 | | 116  64.6  64.9  106  64.6  116  106  64.5 | 6.93  56.42  2.78  8.82  0.01  1.52  1.47  0.03 | | **0.002***  **<.001*****  0.100  **<.001*****  0.922  0.223  0.235  0.857 | |  |
| *Reinstatement* | | | | | | | | |  |
| Stimulus  Block  Group  Stimulus×Block  Block×Group  Stimulus×Group  Stimulus×Block×Group  Sex | | 2  1  1  1.88  1  2  1.88  1 | | 118  63  64.7  104  63  118  104  60.2 | 1.94  38.28  6.97  3.89  0.67  0.42  1.26  1.68 | | 0.148  **<.001*****  **0.010***  **0.026***  0.414  0.660  0.287  0.199 | |  |
| **Valence** | | | | | | | | |  |
| Stimulus  Time  Group  Stimulus×Time  Time×Group  Stimulus×Group  Stimulus×Time×Group  Sex | | 1.55  3.41  1  5.09  3.41  1.55  5.09  1 | | 98  211  65.7  305  211  98  305  61.6 | 67.87  24.28  0.00  37.34  0.95  0.95  1.07  0.61 | | **<.001*****  **<.001*****  0.977  **<.001*****  0.428  0.369  0.377  0.437 | |  |
| **Arousal** | | | | | | | | |  |
| Stimulus  Time  Group  Stimulus×Time  Time×Group  Stimulus×Group  Stimulus×Time×Group  Sex | | 1.49  3.38  1  4.77  3.38  1.49  4.77  1 | | 90.2  218  64.4  286  218  90.2  286  65.2 | 78.72  40.47  2.30  29.77  1.19  0.34  0.51  0.33 | | **<.001*****  **<.001*****  0.134  **<.001*****  0.314  0.651  0.761  0.565 | |  |
| **Fear** | | | | | | | | |  |
| Stimulus  Time  Group  Stimulus×Time  Time×Group  Stimulus×Group  Stimulus×Time×Group  Sex | | 1.41  3.21  1  4.77  3.21  1.41  4.77  1 | | 80.8  204  65.8  272  204  80.8  272  60.5 | 49.53  45.69  3.81  26.95  0.72  1.19  0.74  0.01 | | **<.001*****  **<.001*****  0.055  **<.001*****  0.553  0.297  0.585  0.935 | |  |
| **US expectancy** | | | | | | | | |  |
| Stimulus  Time  Group  Stimulus×Time  Time×Group  Stimulus×Group  Stimulus×Time×Group  Sex | | 1.68  3.28  1  5.04  3.28  1.68  5.04  1 | | 94.9  208  65  309  208  94.9  309  70.5 | 90.22  72.65  1.58  42.08  1.33  0.75  0.62  2.46 | | **<.001*****  **<.001*****  0.213  **<.001*****  0.263  0.454  0.686  0.122 | |  |
| **Compound contingency score** | | | | | | | | |  |
| Group  Sex | | 1  1 | | 64  62.4 | 11.29  1.15 | | **0.001***  0.288 | |  |

* Significant results at *p* < 0.05.

*** Significant results at *p* < 0.001.
